# Health-related quality of life of Australians during the 2020 COVID-19 pandemic: a comparison with pre-pandemic data and factors associated with poor outcomes

**DOI:** 10.1007/s11136-022-03222-y

**Published:** 2022-08-22

**Authors:** Rebecca Mercieca-Bebber, Rachel Campbell, Dayna Jan Fullerton, Sabina Kleitman, Daniel S. J. Costa, Dion Candelaria, Margaret Ann Tait, Richard Norman, Madeleine King

**Affiliations:** 1 NHMRC Clinical Trials Centre, The University of Sydney, Sydney, Australia; 2 Faculty of Medicine and Health, The University of Sydney, Sydney, Australia; 3 Sydney Quality of Life Office, School of Psychology, Faculty of Science, The University of Sydney, Sydney, Australia; 4 School of Psychology, Faculty of Science, The University of Sydney, Sydney, Australia; 5 Susan Wakil School of Nursing and Midwifery, Faculty of Medicine and Health, The University of Sydney, Sydney, Australia; 6 School of Population Health, Curtin University, Perth, WA Australia

**Keywords:** COVID-19, SARSCoV2, Coronavirus, Quality of Life, Population health, Surveys and questionnaires

## Abstract

**Purpose:**

Compare the health-related quality of life (HRQL) of the Australian general population during the COVID-19 pandemic (2020) with pre-pandemic data (2015–2016) and identify pandemic-related and demographic factors associated with poorer HRQL.

**Methods:**

Participants were quota sampled from an online panel by four regions (defined by active COVID-19 case numbers); then by age and sex. Participants completed an online survey about their HRQL [EORTC QLQ-C30 questionnaire and General Health Question (GHQ)], demographic characteristics, and the impact of the pandemic on daily life. HRQL scores were compared to a 2015–2016 reference sample using independent t-tests, adjusted for multiple testing. Associations between 22 pre-specified factors (pandemic-related and demographic) and 15 QLQ-C30 domains and GHQ, were assessed with multiple regressions.

**Results:**

Most domains were statistically significantly worse for the 2020 sample (*n* = 1898) compared to the reference sample (*n* = 1979), except fatigue and pain. Differences were largest for the youngest group (18–29 years) for cognitive functioning, nausea, diarrhoea, and financial difficulties. Emotional functioning was worse for 2020 participants aged 18–59, but not for those 60 +.

All models were statistically significant at *p* < .001; the most variance was explained for emotional functioning, QLQ-C30 global health/QOL, nausea/vomiting, GHQ, and financial difficulties. Generally, increased workload, negative COVID-19 impacts, COVID-19-related worries, and negative attitudes towards public health order compliance were associated with poorer HRQL outcomes.

**Conclusion:**

During the COVID-19 pandemic, Australians reported poorer HRQL relative to a pre-pandemic sample. Risk factors for poor HRQL outcomes included greater negative pandemic-related impacts, poorer compliance attitudes, and younger age.

**Trial registration:**

ANZCTR number is: ACTRN12621001240831. Web address of your trial: https://www.anzctr.org.au/ACTRN12621001240831.aspx. Date submitted: 26/08/2021 2:56:53 PM. Date registered: 14/09/2021 9:40:31 AM. Registered by: Margaret-Ann Tait. Principal Investigator: Madeleine King.

**Supplementary Information:**

The online version contains supplementary material available at 10.1007/s11136-022-03222-y.

## Background

The COVID-19 pandemic, the global response to it, and the associated social and economic impacts have led to it being arguably the most profound and challenging pandemic in history [1]. Australia’s first known cases of COVID-19 were documented on 25 January 2020 in Victoria and New South Wales (NSW) [2]; Australia’s two most populous states. Like most nations internationally, as cases began to rise, Australia sought to minimise local transmission of the virus and the rate of national infection by introducing social distancing orders and restricting local and international travel. Six weeks later, with a national total of 140 cases [3], the Federal Government announced on 13 March 2020 that all mass gatherings of 500 + participants should not take place, and a level three travel warning against non-essential international travel [4]. On 24 March 2020, this escalated to a travel ban and a suspension of non-urgent elective surgery [2], when the national total reached 1709 cases and seven deaths [3].

By mid-late March 2020, all Australian states and territories were under stay-at-home orders or restrictions of some capacity. By 1 April 2020, Australia had 6778 reported cases, which was the 39th highest number of cases per country at that time [5] (acknowledging that access to testing and reporting impacted the comparability of case numbers between countries). A second outbreak commencing in June 2020 affected predominantly the city of Melbourne and parts of regional Victoria [6]. This led to a second, lengthy regional lockdown period in Victoria lasting almost four months.

The COVID-19 pandemic and the associated public health orders undoubtedly had a pervasive impact on Australians’ health, freedoms, and wellbeing. Wilson and Cleary’s model of health-related quality of life (HRQL) acknowledges that all aspects of biological function, symptoms, functional status, general health perceptions, and overall quality of life are uniquely impacted by characteristics of the individual and the environment, respectively [7]. This model aligns with Revicki’s description of quality of life as a multidimensional construct including “physical, psychological, social and somatic domains of functioning and well-being” (p. 888) [8]. The COVID-19 pandemic resulted in some of the most extensive changes to the social environment within Australia in living memory—the impact of which is still being realised. Early international research suggested the pandemic and associated lockdowns and health measures led to increased mental distress and other poor health outcomes [9]. These pandemic-related impacts were independent of pre-existing risk factors for poor mental health, such as being an ethnic minority or being unemployed [9]. It is possible that the pandemic itself may be an independent health trigger, as well as an environmental context, affecting HRQL, within Wilson and Cleary’s model. This is distinct from the impact of COVID-19 infection on HRQL [10].

Given that responses to future pandemics could be managed by targeted responses for key demographic groups, including professions, age groups, or those with pre-existing mental health conditions [11], it is important that we understand how factors related to the “individual” moderate the impact of the pandemic on health outcomes. Similarly, the influence of environmental factors on “functional status” may be mitigated by “social and economic supports” [7], for instance someone who has lost work as a result of lockdown, or who must juggle childcare in addition to working from home, may experience increased stress and poorer outcomes. An understanding of how the COVID-19 pandemic (2020) and associated public health orders impacts the symptoms, functioning, and overall HRQL of everyday Australians is therefore crucial due to the impact these outcomes may place on an already burdened health system, and because these said factors may contribute to an individual’s uptake of health behaviours and risk of infection.

This study had two aims: (1) to describe the impact of the COVID-19 pandemic (2020) and associated public health orders on aspects of the Australian community’s self-reported health, including HRQL, functioning, and other health-related symptoms; and to compare these outcomes to Australian general population reference values collected in 2015–2016; and (2) to identify pandemic-related and demographic factors associated with poorer self-reported health outcomes, in line with Wilson and Cleary’s framework.

## Methods

### Participants and Quota sampling

Australians aged 18–99 years who were members of an online panel managed by the survey and consumer insights company Toluna (https://au.toluna.com) were invited to participate. Sampling was managed by the survey host company, Survey Engine [12]. Quota sampling was conducted at two levels: by region/pandemic intensity and then by age and sex. At the first level, participants were quota sampled by four regions that we defined according to the number of active cases regionally and the relative restrictiveness of prevailing public health orders at the time of the survey, noting that Melbourne and greater Victoria were experiencing a second wave of the pandemic and a long and strict lockdown period in the lead-up to, and during, data collection for this study (see Online Appendix 1). The following four regions of pandemic intensity were defined: (1) Melbourne, the capital city of Victoria: the greatest number of cases and the most restrictive public health orders, including city-wide lockdown; (2) Regional Victoria: the second highest number of cases and the second most restrictive public health orders; (3) NSW and Queensland: some localised hot-spots and border closures; (4) Western Australia, South Australia, Tasmania, the Australian Capital Territory, and the Northern Territory: very few cases, least restrictive public health orders. Note that Australian states are governed by state governments and the two territories are under federal governance; therefore, the nature and timing of specific public health orders and restrictions differed between states and territories. A summary of these is presented in Online Appendix 1.

The second level of quota sampling (by age and sex) occurred within each region, which ensured proportions corresponded to the Australian general population within each region and therefore for the overall sample [13]. Responses to each item were mandatory. Participants who successfully completed the survey were awarded ‘panel points’ by Toluna, which are used to redeem vouchers or consumer goods, with an approximate value of less than 1AUD.

### Survey

Participants completed an online survey about the impact of the COVID-19 pandemic on their daily life, work and study, and about their current health status, HRQL, and mental health. Participants also completed questions about their demographic characteristics. Relevant to this analysis, the surveys completed were:

- *COVID Worry Scale* (21 items)* which contains four subscales: Personal/Family Concerns; Personal Financial Concerns; Economy/Liberties Concerns; Infrastructure/Supplies Concerns and asks how the participant is feeling now. Higher scores indicate higher worry [14].
- *COVID Impact Index* (19 items)* which generates five composite scores based on exploratory factor analysis: job/financial security, routine, mental health; physical health; family responsibilities; alcohol/substance use; loneliness and time. The response timeframe is since the COVID-19 pandemic began. The relative composites and scoring are provided in Online Appendix 2.
- A list of possible impacts of COVID*, both positive and negative, which included the need to home-school dependent children, loss of work, isolation from family members, time to focus on health, impact on study, and increased time with family. The response timeframe is since the COVID-19 pandemic began. For this analysis, the number of impacts endorsed by each participant were summed.
- Attitude and Motivation towards compliance* (9 items) which captures attitudes towards the COVID-19 pandemic and associated health orders. A mean score was calculated with higher scores indicating more positive attitudes and greater willingness to comply.
- *Kessler Psychological Distress scale* [15] (10 items) measuring distress in the past 30 days with a total score.
- *EORTC QLQ-C30 v3* [16] (30 items) a HRQL questionnaire commonly used in cancer clinical studies measuring five functioning domains (physical, role, emotional, social, cognitive), eight symptoms (fatigue, nausea/vomiting, pain, dyspnoea, sleep disturbances, appetite loss, constipation, diarrhoea), financial impact, and global quality of life (see comparator sample section below). We scored these 15 domains according to the QLQ-C30 scoring manual [17]. The response timeframe is the past week. Items do not mention cancer and the domains are generally applicable to all individuals; therefore, comparing QLQ-C30 responses pre- and during the COVID-19 pandemic would provide relevant insight into the effects of the pandemic on health-related domains.
- *General Health Question (GHQ)* “In general would you say that your health is: Excellent/Very good/Good/Fair/Poor?”
- *Demographics* including type of work, living arrangements, marital status, number of children, country of birth, Aboriginal or Torres Strait Islander status, education, age, and sex.

*measures currently undergoing validation. See details and references in Online Appendix 2.

### Comparator sample

Between March 2015 and February 2016, we collected QLQ-C30 reference value data from the Australian general population (*N* = 1979), representative by age and sex, to facilitate interpretation of QLQ-C30 data from Australians with cancer [18]. We also collected Kessler distress scale and GHQ data in the same online survey.

### Data cleaning

We imposed seven quality checks, including unreasonable completion time (completion in less than 30% of the sample’s median completion time, here 7.79 min), and six checks for inconsistent responses, to ensure our final dataset included high-quality and genuine responses, as recommended for social science surveys [19]**.** Participants who failed two or more quality checks or completed in less than 7.79 min were excluded from analysis. Online Appendix 3 describes the quality checks used and includes a flowchart of participant inclusions and exclusions.

### Sample size

We required a minimum sample size of *n* = 1456 for the primary study endpoint, allowing us to detect clinically “small” differences in any of the QLQ-C30 scales [20] with 80% power and a Bonferroni adjusted alpha of 0.003 (Bonferroni adjustment is conservatively recommended for sample size calculation when using the Hochberg method) [21]. Our recruitment target was increased to *n* = 2000 (*n* = 500 per sampling region) to allow for drop out and planned secondary analyses.

### Analysis

To address Aim 1, we calculated the mean and standard deviation of each scale within the QLQ-C30, and GHQ, for each age group (18–29, 30–39, 40–49, 50–59, 60–69, and 70 + years) and sex (male, female) according to their scoring manuals [17, 22]. We also calculated the QLQ-C30 27-item summary score [23].

We then compared the mean scores per scale to our 2015/16 reference sample using independent t-tests for each age group and sex. We hypothesised that health outcomes would generally be worse for the 2020 sample for the following functional and symptom QLQ-C30 domains, which were most applicable to a general population sample in the context of the pandemic [7]: role functioning, emotional functioning, social functioning, cognitive functioning, fatigue, sleep disturbances, financial impact, and global HRQL, as well as for the GHQ score. We interpreted the size of the differences in QLQ-C30 scales according to Cocks’ guidelines [20].

To address Aim 2, we conducted a series of simultaneous multiple regressions for each of the 15 QLQ-C30 domains and the GHQ (outcome variables), using the same 22 explanatory variables (see Online Appendix 4). A set of explanatory variables was chosen a priori based on the existing literature and hypotheses that key characteristics of the individual and environment would predict worse HRQL and health outcomes, in line with Wilson and Cleary’s model. These include increased commitments (including workload and carer responsibilities), higher COVID-related worries, more changes to one’s typical routine, recency of lockdown/severity of the COVID-19 situation within region, lower social support and poorer attitude towards restrictions and regulations generally. The explanatory variables were consistent within each regression model (per domain).

The overall model can be summarised by the following equation:

1 $$ {\text{Y}}_{i} = {\text{a }} + \beta_{{1}} {\text{X }} + \, \beta_{{2}} + \, \beta_{{3}} + \, \beta_{{4}} + \ldots \, \beta_{{{22}}} + {\text{e}}, $$

where Y_*i*_ represents the domain of study for a person *i*, e.g. QLQ-C30 overall health/HRQL, a represents the intercept; *β*_1-22_ represents a regression coefficient for each relevant explanatory variable (22 all together), and e represents error.

More details are presented in Online Appendix 5. All assumptions were checked, and with several caveats described below, satisfied. The descriptive statistics including skewness metrics are outlined in Table A5.1 in online Appendix 5. We applied a Bonferroni adjusted alpha of 0.05/16 scales = 0.003 to determine the significance of overall models; this adjustment is conservative given that domains of the QLQ-C30 are known to be correlated [17, 24]. We then applied the Hochberg adjustment method within each model to determine the significance level of individual explanatory variables based on ranked alphas [21].

All analyses were completed in SPSS v27 and all available item-level data from included participants were used.

## Results

The online survey was active from 21 October to 10 November 2020. During this time, all states and territories were experiencing restrictions to house visitors, hospitality, and indoor and outdoor gatherings/activities. These restrictions had largely been relaxed from those seen earlier in 2020, although Victoria’s restrictions were easing from their strictest levels at Stages 3–4, due to their second wave of infections (see online Appendix 1 for a summary of prevailing restrictions).

A total of 2007 participants completed the survey, of which 1898 passed quality checks and were included in the final analysis (479 from Melbourne, 475 from regional Victoria, 468 from NSW and Queensland, 476 from the remaining states and territories). Participant characteristics are included in Table 1 and online Appendix 6.

**Table 1:** Demographic characteristics

Question	Level	Frequency	2015/16 Sample % (or mean [SD])	2020 Sample % (or mean [SD])	Population % (or mean [SD])^a^	Statistic^b,d^	*p* value^d^
Sex	Male	915	49.3	48.2	49.1	*X*^2^ = .54	.46^c^
	Female	983	50.7	51.8	50.9		
Age	18–29 years	376	22.1	19.8	21.1	*X*^2^ = 2.93	.71^c^
	30–39 years	347	17.8	18.3	18.8		
	40–49 years	314	17.7	16.6	16.5		
	50–59 years	309	16.3	16.3	15.6		
	60–69 years	264	13.2	13.9	13.5		
	70 years or older	287	12.9	15.1	14.7		
State^e^	ACT	35	–	1.8	1.7	*X*^2^ = 776.08	< .001
	NSW	292	–	15.4	32.0		
	NT	6	–	0.3	1.0		
	QLD	176	–	9.3	20.1		
	SA	192	–	10.1	7.2		
	TAS	54	–	2.8	2.2		
	VIC	954	–	50.3	25.3		
	WA	189	–	10.0	10.6		
Country of birth	Australia	1461	74.1	77.0	71.7	*X*^2^ = 65.10	< .001
Aboriginal or torres strait islander status	Yes	74	8.3	3.9	2.8	*X*^2^ = 8.90	.003
Marital status	Married (registered)	866	43.5	45.6	48.1	*X*^2^ = 23.29	< .001
	Separated	67	3.0	3.5	3.2		
	Divorced	158	8.3	8.3	8.5		
	Widowed	65	3.6	3.4	5.2		
	Never married	742	27.3	39.1	35.0		
Children < 5 years	0	1634	75.7	86.1	86.4	*X*^2^ = 1.89	.59^c^
	1	192	13.9	10.1	9.6		
	2	64	8.0	3.4	3.7		
	3 or more	8	2.5	.4	.3		
Highest level of education	Year 11 or below	283	16.3	14.9	21.2	*X*^2^ = 190.33	< .001
	Year 12	315	18.5	16.6	15.7		
	Trade certificate	329	0.2	17.3	24.3		
	Diploma	265	0.2	14.0	10.1		
	Bachelor’s degree	489	23.0	25.8	16.0		
	Higher degree	217	0.2	11.4	12.7		
Kessler-10	Low distress: 10–15	909	52.2	47.9	60.8	*X*^2^ = .21	< .001
	Moderate distress: 16–21	332	21.9	17.5	21.9		
	High distress: 22–30	377	17.5	19.9	8.9		
	Very high: 31–50	280	8.1	14.8	4.0		

*ACT* Australian Capital Territory, *NSW* New South Wales, *NT* Northern Territory, *QLD* Queensland, *SA* South Australia, *TAS* Tasmania, *VIC* Victoria, *WA* Western Australia

^a^Age and sex values were obtained from national, state and territory population data, Australian Bureau of Statistics, published June, 2021: https://www.abs.gov.au/statistics/people/population/national-state-and-territory-population/latest-release. Population values for State, Country of Birth, Aboriginal or Torres Strait Islander Status, Marital Status, were obtained from the Australian Bureau of Statistics 2016 Census (note, this data was not limited to those aged 18 and over). Population values for Children < 5 years and Highest Level of Education were derived from the Household, Income and Labour Dynamics in Australia Survey (HILDA, Wave 19), limited to those aged 18 and over. Kessler-10 (measure of psychological distress) values and prevalence of chronic conditions were derived from the Australian Bureau of Statistics National Health Survey, published December, 2018: https://www.abs.gov.au/statistics/health/health-conditions-and-risks/national-health-survey-first-results/latest-release. Population data for other chronic conditions was not directly comparable in format

^b^The chi-squared goodness of fit test was used to compare observed distributions to those expected based on Australian population data

^c^Indicates sample is not statistically significantly different from the Australian general population

^d^Comparisons are between 2020 sample and population data^a^

^e^Data not available from 2015 to 2016 reference sample: Mercieca-Bebber et al. [18]

### Impact of the 2020 COVID-19 pandemic on health-related domains, compared to 2015/16 reference values

Table 2 shows QLQ-C30 and GHQ scores for the 2020 sample compared to a pre-COVID-19 reference sample [18] from 2015 to 2016 by age group and sex. The largest differences seen were in the 18–29 year group, whereby the 2020 sample reported worse cognitive functioning, nausea, diarrhoea, and financial difficulties, with these differences deemed of a medium size according to interpretation guidelines [20]. Most HRQL domains were statistically significantly worse for the 2020 sample, by age group and sex, as compared to the reference sample. Notable exceptions include fatigue, pain and the QLQ-C30 summary score, which were statistically similar in both samples across all age groups. Emotional functioning was statistically significantly worse for the 2020 participants aged 18–59, however there was no difference for older subgroups aged 60 and over.

**Table 2:** Mean QLQ-C30 and GHQ scale scores by age group and sex, comparing the 2020 sample and 2015/16 reference sample

	2020 Sample mean (SD)	Reference value (SD) from 2015/16 sample	df	t	*P*^*a*^	Clinical interpretation of difference^b^
Age group						
18–29						
Global QOL	62.85 (21.53)	66.8 (19.3)	776	2.69	.007*	Trivial
Physical	81.33 (22.05)	88.7 (18.7)	776	5.01	< .001*	Small
Role	73.40 (26.84)	85.4 (25.0)	776	6.44	< .001*	Small
Emotional	66.58 (26.26)	81.9 (24.8)	776	8.35	< .001*	
Cognitive	77.44 (25.21)	87.4 (25.5)	776	5.48	< .001*	Medium
Social	80.23 (26.93)	90.0 (23.6)	776	5.37	< .001*	Small
Fatigue	27.93 (24.60)	25.9 (21.7)	776	− 1.22	.22	Trivial
Nausea	17.42 (24.07)	7.6 (22.0)	776	− 5.93	< .001*	Medium
Pain	21.59 (26.08)	23.1 (23.4)	776	0.85	.40	Trivial
Dyspnoea	19.95 (29.03)	13.9 (24.3)	776	− 3.14	.002*	Small
Insomnia	31.47 (32.92)	25.2 (30.7)	776	− 2.74	.006*	Small
Appetite loss	22.87 (30.16)	13.2 (25.9)	776	− 4.78	< .001*	Small
Constipation	19.41 (30.18)	11.3 (25.7)	776	− 4.02	< .001*	Small
Diarrhoea	18.53 (28.43)	8.6 (25.7)	776	− 5.10	< .001*	Medium
Financial difficulties	18.09 (28.56)	5.4 (24.1)	776	− 6.68	< .001*	Medium
Summary score^c^	76.91 (20.24)	79.23 (17.49)	775	1.70	.09	
GHQ	2.58 (1.02)	2.36 (.94)	775	− 3.12	.002*	
30–39						
Global QOL	62.97 (22.65)	66.2 (21.8)	670	1.88	.06	Trivial
Physical	87.13 (20.12)	88.8 (20.5)	670	1.06	.29	Trivial
Role	81.12 (26.37)	87.6 (24.7)	670	3.29	.001*	Small
Emotional	67.27 (28.27)	80.4 (25.7)	670	6.31	< .001*	
Cognitive	81.80 (22.51)	87.9 (23.9)	670	3.40	.001*	Small
Social	84.73 (25.35)	89.5 (25.4)	670	2.44	.015	Trivial
Fatigue	25.58 (23.53)	25.6 (23.8)	670	0.01	.99	Trivial
Nausea	10.47 (19.16)	7.7 (22.9)	670	− 1.69	.09	Trivial
Pain	17.53 (25.26)	21.3 (24.8)	670	1.95	.05	Trivial
Dyspnoea	13.93 (26.60)	14.4 (25.2)	670	0.24	.81	Trivial
Insomnia	31.22 (33.84)	23.6 (30.8)	670	− 3.05	.002*	Small
Appetite loss	14.31 (23.16)	12.8 (25.3)	670	− 0.81	.42	Trivial
Constipation	12.78 (23.78)	11.1 (25.7)	670	− 0.88	.38	Trivial
Diarrhoea	10.57 (22.03)	8.0 (24.2)	670	− 1.44	.15	Trivial
Financial difficulties	13.54 (27.28)	8.4 (27.8)	670	− 2.42	.02	Small
Summary score	81.97 (17.71)	79.77 (18.35)	669	− 1.58	.11	
GHQ	2.69 (1.02)	2.53 (1.01)	669	− 2.04	.04	
40–49						
Global QOL	62.69 (21.28)	68.1 (21.7)	634	3.17	.002*	Small
Physical	87.18 (20.49)	92.3 (17.6)	634	3.38	.001*	Small
Role	84.13 (23.58)	89.8 (24.4)	634	2.98	.003*	Trivial
Emotional	67.70 (28.71)	79.0 (25.5)	634	5.24	< .001*	
Cognitive	80.15 (25.18)	87.3 (23.3)	634	3.71	< .001*	Small
Social	84.50 (25.81)	89.7 (26.3)	634	2.52	.01*	Small
Fatigue	25.12 (25.36)	24.1 (23.0)	634	− 0.53	.60	Trivial
Nausea	8.86 (16.75)	3.1 (13.3)	634	− 4.80	< .001*	Small
Pain	20.28 (28.19)	21.7 (27.0)	634	.65	.52	Trivial
Dyspnoea	12.53 (23.66)	9.5 (21.5)	634	− 1.69	.09	Trivial
Insomnia	31.10 (34.21)	25.6 (32.9)	634	− 2.07	.04	Small
Appetite loss	12.63 (22.6)	7.0 (19.9)	634	− 3.33	.001*	Small
Constipation	13.80 (25.15)	7.9 (20.1)	634	− 3.26	.001*	Small
Diarrhoea	10.72 (20.85)	6.0 (18.3)	634	− 3.03	.003*	Small
Financial difficulties	16.14 (28.97)	6.8 (25.3)	634	− 4.33	< .001*	Small
Summary score	82.20 (17.82)	82.11 (16.14)	634	− 0.07	.95	
GHQ	2.80 (1.03)	2.88 (.95)	634	1.02	.31	
50–59						
Global QOL	61.68 (22.45)	69.0 (23.3)	603	3.93	< .001*	Small
Physical	86.49 (18.92)	90.1 (19.1)	603	2.33	.02	Trivial
Role	85.71 (22.23)	91.1 (22.3)	603	2.98	.003*	Trivial
Emotional	73.00 (24.55)	80.0 (24.0)	603	3.55	< .001*	
Cognitive	84.41 (20.73)	88.1 (21.3)	603	2.16	.03	Small
Social	87.11 (23.95)	89.4 (25.1)	603	1.15	.25	Trivial
Fatigue	21.97 (21.25)	21.3 (23.3)	603	− 0.37	.71	Trivial
Nausea	4.47 (12.84)	2.2 (11.2)	603	− 2.32	.02	Trivial
Pain	23.57 (28.88)	21.9 (28.1)	603	− 0.72	.47	Trivial
Dyspnoea	10.36 (21.33)	8.9 (23.5)	603	− 0.80	.42	Trivial
Insomnia	31.93 (33.03)	25.1 (30.1)	603	− 2.66	.01	Small
Appetite loss	9.06 (19.84)	6.1 (20.1)	603	− 1.82	.07	Trivial
Constipation	10.57 (22.22)	8.1 (21.2)	603	− 1.40	.16	Trivial
Diarrhoea	6.80 (16.36)	2.5 (13.6)	603	− 3.52	< .001*	Small
Financial difficulties	10.25 (21.47)	7.3 (25.9)	603	− 1.52	.13	Trivial
Summary score	84.44 (14.60)	83.69 (15.13)	603	− 0.40	.69	
GHQ	3.03 (.99)	2.94 (1.05)	603	− 1.08	.28	
60–69						
Global QOL	62.18 (21.74)	70.8 (21.1)	503	4.52	< .001*	Small
Physical	85.20 (19.60)	89.4 (16.6)	503	2.61	.01	Trivial
Role	87.18 (22.16)	91.5 (20.6)	503	2.27	.02	Trivial
Emotional	77.11 (24.85)	82.0 (17.7)	503	2.56	.01	
Cognitive	87.50 (19.27)	88.0 (15.8)	503	0.32	.75	Trivial
Social	88.83 (20.56)	94.5 (19.5)	503	3.18	.002*	Small
Fatigue	21.00 (21.14)	21.6 (19.2)	503	0.33	.74	Trivial
Nausea	3.66 (10.44)	1.9 (7.3)	503	− 2.21	.03	Trivial
Pain	24.87 (30.17)	21.1 (26.0)	503	− 1.51	.13	Trivial
Dyspnoea	10.86 (20.35)	10.4 (18.9)	503	− 1.43	.15	Trivial
Insomnia	28.03 (32.19)	24.9 (28.0)	503	− 1.17	.24	Trivial
Appetite loss	6.69 (15.70)	4.0 (14.0)	503	− 2.04	.04	Trivial
Constipation	7.07 (19.07)	6.5 (18.2)	503	− 0.34	.73	Trivial
Diarrhoea	6.06 (15.28)	4.4 (13.9)	503	− 1.28	.20	Trivial
Financial difficulties	6.69 (13.51)	3.8 (16.4)	503	− 2.15	.03	Trivial
Summary score	85.97 (13.51)	86.09 (11.03)	503	0.11	.91	
GHQ	3.08 (.97)	2.97 (.97)	503	− 1.27	.20	
70 and over						
Global QOL	65.16 (21.56)	72.3 (21.6)	520	3.76	< .001*	Small
Physical	81.30 (21.18)	84.6 (19.9)	520	1.83	.07	Trivial
Role	83.86 (23.17)	89.2 (21.4)	520	2.73	.01	Trivial
Emotional	82.38 (20.02)	82.8 (17.8)	520	0.25	.80	
Cognitive	88.56 (17.35)	90.2 (12.6)	520	1.25	.21	Trivial
Social	86.30 (23.53)	92.8 (20.2)	520	3.40	.001*	Small
Fatigue	23.34 (22.89)	23.5 (18.1)	520	.09	.93	Trivial
Nausea	1.92 (7.58)	3.0 (7.7)	520	1.61	.11	Trivial
Pain	24.33 (28.04)	20.8 (27.7)	520	− 1.44	.15	Trivial
Dyspnoea	16.14 (25.22)	12.2 (22.2)	520	− 1.90	.06	Trivial
Insomnia	21.37 (27.74)	20.7 (24.4)	520	− 0.29	.77	Trivial
Appetite loss	5.92 (17.40)	4.9 (15.0)	520	− 0.72	.47	Trivial
Constipation	6.27 (16.94)	10.3 (19.3)	520	2.51	.01	Trivial
Diarrhoea	3.95 (12.46)	3.8 (12.8)	520	− 0.13	.89	Trivial
Financial difficulties	6.16 (16.16)	4.2 (17.1)	520	− 1.34	.18	Trivial
Summary score	86.09 (13.79)	85.17 (11.19)	520	− 0.84	.40	
GHQ	3.06 (.98)	2.94 (.98)	520	− 1.39	.16	
Sex						
Males						
Global QOL	63.33 (22.00)	66.9 (21.2)	1810	3.52	< .001*	Trivial
Physical	84.76 (20.97)	86.6 (19.7)	1810	1.93	.05	Trivial
Role	82.33 (23.89)	84.6 (24.5)	1810	2.00	.05	Trivial
Emotional	75.66 (24.92)	83.2 (23.7)	1810	6.60	< .001*	
Cognitive	83.93 (21.86)	86.7 (22.3)	1810	2.67	.01	Trivial
Social	85.01 (25.18)	86.5 (24.9)	1810	1.27	.21	Trivial
Fatigue	21.60 (21.72)	23.6 (21.6)	1810	1.97	.05	Trivial
Nausea	8.09 (17.56)	6.1 (17.4)	1810	− 2.42	.02	Trivial
Pain	20.71 (27.41)	24.3 (25.7)	1810	3.27	.001*	Trivial
Dyspnoea	14.94 (25.91)	14.5 (23.9)	1810	− 0.38	.71	Trivial
Insomnia	25.76 (30.89)	25.5 (29.3)	1810	− 0.18	.85	Trivial
Appetite loss	12.02 (22.57)	9.3 (21.9)	1810	− 2.60	.01	Trivial
Constipation	11.22 (23.24)	10.0 (22.6)	1810	− 1.13	.26	Trivial
Diarrhoea	10.64 (21.68)	5.8 (20.8)	1810	− 4.85	< .001*	Small
Financial difficulties	13.08 (25.34)	9.1 (24.0)	1810	− 3.43	.001*	Small
Summary score	83.59 (17.23)	82.56 (16.64)	1810	− 1.29	.20	
GHQ	2.76 (1.04)	2.65 (1.04)	1810	− 2.25	.02	
Females						
Global QOL	62.54 (21.77)	70.1 (21.7)	1905	7.59	< .001*	Small
Physical	84.75 (20.28)	91.7 (18.2)	1905	7.89	< .001*	Small
Role	81.89 (25.53)	92.8 (22.3)	1905	9.95	< .001*	Small
Emotional	68.20 (27.17)	78.7 (24.2)	1905	10.06	< .001*	
Cognitive	81.96 (23.00)	89.3 (21.6)	1905	7.19	< .001*	Small
Social	84.99 (24.39)	94.7 (22.8)	1905	8.99	< .001*	Small
Fatigue	27.01 (24.54)	24.2 (22.4)	1905	− 2.61	.009	Trivial
Nausea	8.70 (17.49)	3.2 (16.5)	1905	− 7.07	< .001*	Small
Pain	22.86 (27.99)	19.4 (26.3)	1905	− 2.78	.005*	Trivial
Dyspnoea	13.53 (24.19)	9.0 (22.1)	1905	− 4.27	< .001*	Small
Insomnia	32.82 (33.88)	23.2 (30.7)	1905	− 6.50	< .001*	Small
Appetite loss	13.02 (23.88)	7.9 (21.8)	1905	− 4.89	< .001*	Small
Constipation	12.95 (24.89)	8.8 (22.7)	1905	− 3.81	< .001*	Trivial
Diarrhoea	9.26 (20.44)	6.0 (19.4)	1905	− 3.57	< .001*	Small
Financial difficulties	11.50 (24.26)	3.3 (23.8)	1905	− 7.45	< .001*	Small
Summary score	81.66 (16.78)	81.92 (14.90)	1904	.36	.72	
GHQ	2.94 (1.00)	2.81 (.98)	1904	− 2.87	.004*	

*GHQ* general health question from the SF-36 questionnaire

^a^We applied a Hochberg adjustment to *p* values to determine statistical significance

^b^Thresholds for clinical meaningfulness of differences in QLQ-C30 scales have been determined by Cocks’ guidelines: Cocks et al. [^20^] (which excludes emotional functioning and the summary score)

^c^The EORTC QLQ-C30 summary score is calculated based on the mean (all converted to a uniform scale direction) of 13 of the 15 QLQ-C30 scales (excluding the Global Quality of Life and Financial Impact scales)

*p < adjusted significance level

The 2020 sample’s emotional functioning scores were substantially lower than the reference sample, with the difference ranging from 7 to 15 points, with larger differences amongst the younger age groups.

GHQ scores were only statistically different between pre-pandemic and 2020 samples for the 18–29 age group, with the 2020 sample reporting worse general health. The global health/HRQL scale of the QLQ-C30 was statistically significantly worse for all age groups except 30–39 years, during the pandemic. For those aged 40–70 +, differences were considered “small”. For the 18–29 and 30–39 year old, the 2020 sample reported trivially worse global health/HRQL on average, as compared to the reference sample.

Interestingly, amongst men, pain and fatigue were statistically significantly better in the 2020 sample, although the sizes of these differences were considered trivial [20]. Amongst women, all scales, apart from the QLQ-C30 summary score, were statistically significantly worse in the 2020 sample. All QLQ-C30 differences were considered small, apart from fatigue, pain, and constipation, which were trivial according to Cock’s interpretation guidelines [20].

### Associations between perceived COVID-19 related impacts and health-related domains

Descriptive statistics and correlations for all variables in the regression models are provided in Appendix 5. All models met the assumption of linearity. GHQ, global health/HRQL, and emotional functioning scales met the assumption of normality of the residuals. All other domains did not satisfy the assumptions of normality of the residuals and homoscedasticity. This may have been caused by these variables having skewed distributions (negatively for functioning domains and positively for symptoms, as observed via histograms and skewness statistics reported in Appendix 5). However, these distributions are expected of a non-clinical population. Deviations from normality of the residuals are less serious in large samples; therefore, no transformations were applied [25]. There were no indications of major multicollinearity issues, with Tolerance statistics ranging between .42 and .94 and variance inflation factor (VIF) estimates between 1.02 and 2.47 [26, 27]. Table 3 shows a summary of 16 regression models (see Eq. 1). It includes estimates of variance predicted for the overall model (R^2^) with the relevant F- and p-values and standardised regression coefficients (betas). More details are provided in Appendix 5.

**Table 3:** Summary of regression models examining relationships between EORTC QLQ-C30 scales and COVID-19 related impacts

Overall model	Outcome variable	Global health/QOL	Physical function	Role function	Emotional function	Cognitive function	Social function	Fatigue	Nausea/vomiting	Pain	Dyspnoea	Insomnia	Appetite loss	Constipation	Diarrhoea	Financial difficulties	GHQ
Variance explained (R^2^)	Overall variance explained	22.5%	15.7%	15.2%	35.6%	17.3%	16.4%	15.5%	18.8%	10.7%	11.1%	16.6%	15.4%	10.7%	10.9%	17.9%	18.7%
F (22,1871) and *p* values	Overall model	24.75, *p* < .001	15.82, *p* < .001	15.25, *p* < .001	46.96, *p* < .001	17.79, *p* < .001	16.63, *p* < .001	15.59, *p* < .001	19.65, *p* < .001	10.19, *p* < .001	10.59, *p* < .001	16.97, *p* < .001	15.54, *p* < .001	10.16, *p* < .001	10.38, *p* < .001	18.55, *p* < .001	19.54, *p* < .001
*Explanatory variables and standardised beta coefficients*																	
COVID-19 related impacts	COVID Impact Index: better job security, finances, routine, mental health & relationships	.17**	− .08*	.03	.15**	.05	.07	− .01	.05	.03	.08*	− .03	.01	− .01	.13**	.04	− .10**
	COVID Impact Index: better physical health/sleep/nutrition	.19**	.07	.04	.11**	.13**	.09**	− .14**	− .02	− .06*	− .06*	− .19**	− .08*	.00	− .06*	− .09**	− .24**
	COVID Impact Index: less family responsibilities	− .09**	− .03	.03	− .06	− .03	− .01	.01	.00	.02	.02	− .03	.00	.07*	.02	− .03	.05*
	COVID Impact Index: more alcohol/substance use	− .01	.01	− .03	− .04	− .03	− .05	.01	.02	− .01	.03	.04	.02	.01	.00	.03	− .02
	COVID Impact Index: more lonely/bored	− .09**	− .02	.02	− .10**	− .06*	− .08**	.08**	.01	.08**	.01	.08**	.04	.05*	.01	.06	.06
	COVID Worry scale: Personal Financial Concerns	− .02	.10**	.03	− .18**	− .08*	.06	.02	.01	− .02	− .05	.11**	.04	.03	.12**	.12**	.03
	COVID Worry scale: Personal/Family Concerns	− .22**	− .22**	− .18**	− .29**	− .16**	− .19**	.20**	.12**	.17**	.18**	.16**	.16**	.20**	.07	.17**	.19**
	COVID Worry scale: Economy/Liberties Concerns	.04	.05	.04	− .00	.03	.01	.01	− .07	− .04	.01	.00	− .07*	− .07*	− .08	− .09**	− .03
	COVID Worry scale: Infrastructure/Supplies Concerns	− .02	− .17**	− .12**	.02	− .04	− .16**	.06	.16**	.10*	.11**	− .03	.10*	.04	.06	.11**	− .03
	Number of positive impacts	.12**	.07*	.05	.05	.02	.04	− .06*	− .03	− .08**	− .04	− .03	.00	.04	− .04	− .02	− .09**
	Number of negative impacts	− .09**	− .13**	− .14**	− .10**	− .14**	− .12**	.11**	.08**	.16**	.12**	.10**	.07*	.06	.11**	.15**	.04
	Number of other impacts	.06	.04	− .02	.00	.04	.03	− .01	.01	− .06*	− .02	.01	− .05	− .01	.00	− .04	− .08*
	Attitude and Motivation towards compliance—total score	.13**	.20**	.11**	.08**	.11**	.13**	− .06*	− .19**	− .06*	− .16**	− .06	− .12**	− .17**	− .15**	− .15**	.01
	Healthcare services worker	.03	.05*	.04*	− .01	.01	.02	− .03	− .03	− .04*	− .04	.01	− .06*	.00	− .02	− .01	− .02
	Change in work hours (before first lockdown—now)	.01	.06*	.03	.01	− .02	.04	− .03	− .02	− .05*	− .02	− .02	.01	.01	− .01	− .02	− .01
	Cared for children who were usually at school/day care whilst working	− .01	− .01	.01	.02	− .01	.02	− .04	− .03	.00	− .03	− .01	− .01	− .01	− .02	− .02	.03
Location/severity of COVID− 19 situation^a^	Melbourne VIC metro	.06*	.05*	− .01	.07**	.07*	.08**	− .08*	− .11**	− .09**	− .07*	− .09**	− .11**	− .07*	− .05	− .09**	− .07*
	VIC regional	.01	.01	− .04	.07	.02	.03	− .02	− .06*	.01	− .03	− .01	− .02	− .02	− .02	− .03	− .03
	NSW/QLD	.04	− .02	− .03	.07**	.04	.01	.00	− .06*	− .02	− .01	.00	− .04	− .01	− .06*	− .03	− .01
Demographic variables	Living with partner	.08**	.07**	.08**	.04	.02	.04	− .05*	.01	− .06*	− .05*	− .04	− .03	.01	.00	− .02	− .04
	Age	− .09**	− .12**	.02	.06	.05	− .01	.00	− .17**	.13**	.03	.03	− .13**	− .08*	− .10**	.00	.21**
	Sex	.01	.00	.00	− .10**	− .02	.02	.09**	.03	.02	− .03	.08**	.01	.03	− .02	− .04	.06*

*p < .05

**p < adjusted significance level

^a^Locations within Australia are indicative of the severity of the COVID-19 situation at the time of survey. See online Appendix 1

All models were statistically significant at *p* < .001. The models that explained most of the variance in the distribution were emotional functioning (35.6%)), QLQ-C30 global health/HRQL (22.5%), Nausea/vomiting (18.8%), GHQ (18.7%), and financial difficulties (17.9%). Observation of zero-order correlations between explanatory variables and dependent variables (see Tables A5.2–A5.4 in online Appendix 5) suggest that 10 of the 95 *significant* beta coefficients (or 2.8% of the 352 coefficients reported in Table 3 in total) might have been due to a suppression effect. This occurs when an independent variable (IV; the suppressor) either correlates positively with another IV and negatively with the dependent variable (DV), or vice versa, or serves to control for (suppress) variance in the IV which is irrelevant to the DV [27]. The suppressor variable will increase the regression weight of the IV it is correlated with [27]. Thus, if a beta coefficient is high and the correlation is low, this signals a suppression effect [27].

Better emotional functioning was most strongly associated with lower personal/family concerns, lower financial concerns, better job security, finances, mental health, and social life due to pandemic-related health orders, better physical health/activity, sleep, and nutrition, having less loneliness, fewer perceived negative impacts of the COVID-19 pandemic, more positive compliance attitudes, being male, and living in NSW or Queensland. Living in Melbourne was also significant though possibly due to a suppression effect, so this finding should be interpreted with caution.

Better global health/HRQL was most strongly associated with less worry about self or family being infected with COVID-19, positive impacts on work/finances, mental health and relationships, positive impacts on physical health and activity, less family responsibilities, more positive impacts of lockdown/regulations and less negative impacts, positive attitudes towards compliance, and living with partner. Less loneliness and time, and younger age were also significant, albeit likely due to a suppressor effect.

More nausea/vomiting was most strongly associated with worries about self or family, worries about infrastructure and supplies, negative attitudes towards compliance, younger age, and not living in Melbourne.

The factors most strongly associated with a poorer (higher) GHQ score included a more negative impact of the COVID-19 pandemic on physical health/activity, job security/finances/mental health, personal/family concerns, fewer number of positive and other impacts, and older age.

Greater financial difficulties were most strongly associated with all aspects of COVID-19-related worry, worse impact of the COVID-19 pandemic on physical health, higher number of negative impacts of the COVID-19 pandemic, negative attitudes towards compliance with public health orders and not living in Melbourne.

Further details on the results for all scales, including reliability estimates, are provided in online Appendix 5.

## Discussion

Our study found that members of the Australian general population reported significantly worse HRQL outcomes during the COVID-19 pandemic as compared to general population data collected four years earlier. On average, women in the 2020 pandemic sample reported poorer HRQL on all domains as compared to women in the pre-COVID-19 reference sample collected in 2015/16. Men in the 2020 sample experienced poorer HRQL than men in the 2015/16 sample for most domains, with the exception of pain, which was somewhat surprisingly better in the 2020 sample; and dyspnoea, insomnia, and constipation, for which there was no difference. The youngest participants in our 2020 sample (18–29 years) appeared to be most affected by the circumstances imposed by the COVID-19 pandemic. On average they scored worse than the 2015/16 sample for all domains, and although many of these differences were relatively small; differences in emotional and cognitive functioning, nausea, and financial difficulties were in the medium size range. Furthermore, differences in emotional functioning between the 2020 and the 2015/16 reference sample were larger in value for the younger age groups than for the older age groups.

When we explored pandemic-related factors associated with these health domains, we found that all models were statistically significant, with certain domains having a higher percentage of the sample variance accounted for by our 22 explanatory variables. Notably, emotional functioning had over a third (36%) of its variance explained by pandemic-related variables. This finding aligns with the results from our first aim, showing large point differences in scores in 2020 compared to 2015/16 for emotional functioning, particularly for younger Australians.

About a fifth of the variance in global health/HRQL was explained by pandemic-related explanatory variables. A similar pattern emerged for financial difficulties and nausea/vomiting. The results for emotional functioning and global health/ HRQL are unsurprising, if we return to Wilson and Cleary’s model for HRQL [7], which explains that characteristics of the environment (in this case, the COVID-19 pandemic and associated restrictions/lockdowns) can moderate all aspects of the HRQL pathway, and possibly act as an independent health trigger. For the majority of Australians, the changes to everyday life that accompanied the pandemic would have placed great strain on their daily routines, freedoms, and connections with their communities and the world. New challenges called for a need for adaptations, such as working from home and learning to use communications technology to stay in touch with colleagues, friends, and family members outside the home. Many Australians experienced flow-on challenges and pressures, such as a need to juggle work responsibilities in addition to caring for young children and home-schooling school-aged children. Some lost the opportunity to work completely due to the nature of their jobs, bringing financial duress to themselves and their dependents.

The results of our model for emotional functioning suggest the negative impact of these challenges. Those who were more worried about themselves and their family, had more financial concerns, a worse work and social life (including virtually), and more negative impacts of the COVID-19 pandemic in general had worse emotional functioning. Mental health was sign-posted as an urgent research priority during the pandemic, as experts anticipated that the combination of pandemic conditions (e.g. lockdown, media coverage, and health messages) would negatively impact mental health, particularly for vulnerable groups [28], which appears to be evident in this sample mainly amongst the younger participants. Indeed, other studies have reported high levels of pandemic-related stress amongst young people [29]. A similar detriment in emotional functioning as a result of the 2020 COVID-19 pandemic in Spain was observed, as compared with pre-pandemic (2019) Spanish population data using the same questionnaire *p* < .001 [30]. It should be noted, however, that a meta-analysis reported heterogenous findings regarding the psychological impact of the COVID-19 pandemic [31]. The results of the model for nausea/vomiting may reflect the somatic impacts of poorer emotional functioning, or perhaps an effect of substance use. An US study reported increases in anxiety and depression, as well as increases in use of substances to feel better. In that study, 22% reported an increase in alcohol intake, and 14% reported an increase in use of marijuana [32]. Further research would be needed to examine these links in detail.

Better overall health and global health/HRQL (GHQ and QLQ-C30, respectively) were associated with less worries about infection, work and family responsibilities, more positive impacts of COVID-19; greater willingness to comply; and living with a partner. These results seem to reflect a positive impact of feeling supported by, and connected to, others as well as a negative impact of additional responsibilities, particularly where families are concerned. Similar results were seen in a Sydney-based survey of older Australians, conducted towards the end of the first wave of infections in May 2020 [7].Those who reported better emotion regulation strategies, higher engagement with family or friends, and use of new technologies to support communication experienced better emotional health and HRQL [7]. Our findings suggest that personal circumstances and propensities are very important in buffering or amplifying the impact of pandemics, in line with the role of individual and environmental characteristics within Wilson and Cleary’s model for HRQL.

The observed differences in the financial difficulties of Australians during the pandemic are also unsurprising. Australians with greater financial difficulties also reported higher COVID-19-related worry, a higher number of negative impacts of the COVID-19 pandemic and negative attitudes towards compliance. The Australian government offered support to those who lost work and income through various schemes. The most significant of these schemes was the Job Keeper Payment of $650–1200AU per fortnight [33], which ended on 28 March 2021. However, the payment may have been less than many of these individuals earned per week (comparator data is not available), and may not have compensated for long-term loss of business or income beyond the end of the scheme. Furthermore, the loss of social interaction and mental stimulation one experiences in the workplace, likely also impacted the mental health of those who were unable to work.

Only one domain, fatigue, behaved differently to what we hypothesised. We expected fatigue would be higher as a result of the added responsibilities placed on participants due to increased work and caregiving responsibilities, however fatigue was unchanged in most age groups. One possible explanation is that participants benefited from working from home, saved time from not commuting to the office, and had more work-life balance including more opportunity for exercise and leisure activities. For some, the saved time may have been spent catching up on sleep. Exercise is known to reduce fatigue [34]. Exercise was commonly allowed as an essential reason for leaving the house, which may have offered consequent benefits to reduce fatigue. Interestingly, fatigue was also lower in the Spanish study amongst the 2020 sample compared to pre-pandemic sample (2019) [30].

### Strengths and limitations

Strengths of our study include the ability to compare our 2020 data to reference data from 2015 to 2016 across all domains to demonstrate change since the pandemic. Like our pandemic sample, our reference sample was recruited from an online panel and data collected via online survey. Other strengths include the large sample size, and the use of validated self-report measures.

Our survey was conducted towards the end of the 2020 lockdown period, where most Australian states and territories had eased restrictions (see online Appendix 1) and case numbers were lower. Australia benefited from strict lockdown orders and high rates of compliance, through significantly fewer COVID-19 infections in 2020 compared to other countries [5]. A study on the impact of policies on social distancing behaviours in the USA showed that state-wide stay-at-home orders and limits on hospitality were the strongest measures to reduce mobility in the community [35], and overall infection rates. It is possible that Australians recognised the success of 2020 lockdown compared to international infection rates, which may have impacted their survey responses. As a consequence, our estimates of the impact of the pandemic are likely smaller than we would have observed if we had collected data at the peak time of infections.

Another possible limitation is the use of a cancer-specific HRQL measure, the QLQ-C30, to assess health outcomes amongst the general population. We do not feel this has impacted our results, as the questions do not refer specifically to cancer, the symptoms and functional concerns are general in nature and known to everyday people. In fact, the use of the QLQ-C30 questionnaire could be seen as a strength, as it helped us to identify an unexpected impact of the pandemic on nausea and vomiting that we may not otherwise have seen. It was used similarly in a Spanish study [30]. We also used guidelines for interpreting the clinical significance of differences to interpret the differences between mean scores of our 2020 data compared to our 2015/16 data—both obtained from non-clinical samples. Cock’s guidelines do not provide effect size estimates for the emotional functioning domain [20].

We acknowledge that despite our use of quota sampling to ensure adequate representation of age and sex, which are key variables for HRQL in the general population given our chosen HRQL measure [36], our sample was not representative on other variables as shown in Table 1; therefore, readers must interpret results accordingly.

Finally, a small number of significant effects were likely the result of a suppressor effect [27]. Whilst it is difficult to hypothesise the presence of the suppressor effect a priori [27], these results may inform future studies examining similar issues. Importantly, despite utilising 16 simultaneous regression models with 22 identical explanatory variables, only a very small percentage of effects (less than 3%) were likely to be the result of a suppressor effect. An additional strength of this research is that no multicollinearity issues were detected within each model and the significance levels were adjusted based on Hochberg’s procedure [20].

### Future studies

It will be interesting to conduct further analyses to determine whether specific subgroups of the Australian population were differentially affected for certain domains—such as essential workers, front-line workers, parents of school-aged children, people without work, people with family abroad, residents of aged care facilities, and other vulnerable groups. Examining the impact of the 2021 lockdowns due to COVID-19 Delta variant outbreaks in Eastern Australia on self-reported outcomes as compared to our 2020 data would also be of interest. The 2021 outbreak occurred in a climate where many Australians were fatigued by repeated, long lockdown periods [37]. In early 2021, Australia gained access to COVID-19 vaccines. Uptake was initially slow, due to supply shortages and some community reluctance, however by mid-October 2021, 55% of the Australian population was fully vaccinated, compared to 36% internationally, and by December 2021, 76.9% was fully vaccinated [38]. Therefore, there are key differences and challenges that may have impacted the Australian population since our 2020 survey. Finally, international comparisons, and historical comparisons (to past pandemics) [1] would be of interest to a global audience.

### Conclusion

In conclusion, our survey results suggest that the COVID-19 pandemic and associated restrictions and health orders likely impacted most heath domains—with larger differences observed amongst younger adults and women. Domains most strongly associated with pandemic-related variables were emotional functioning, general health, financial difficulties, and nausea and vomiting—although all health domains were statistically significantly associated with pandemic-related factors. Our data could be used to identify Australian sub-groups most at risk of poor health outcomes in the event of future pandemics. This information could be used to develop and target interventions to mitigate these risks or improve outcomes.

## Supplementary Information

Below is the link to the electronic supplementary material.

Supplementary file1 (PDF 1269 KB)

## Data Availability

Subject to author approval and following publication of final paper, de-identified individual participant data underlying published results may be available only to researchers who provide a methodologically sound proposal.

## Code availability

As above.
