# Supplementary material for: Health-related quality of life of Australians during the 2020 COVID-19 pandemic: a comparison with pre-pandemic data and factors associated with poor outcomes
Source: Qual Life Res. 2022 Aug 22;32(2):339–55. doi: 10.1007/s11136-022-03222-y (PMC9393100; doi:10.1007/s11136-022-03222-y)
Supplement: Supplementary file 1 — Supplementary file1 (PDF 1269 KB) [file 11136_2022_3222_MOESM1_ESM.pdf]

Supplementary appendix for the manuscript:

Mercieca-Bebber R, Campbell R, Fullerton DJ, Kleitman S, Costa DSJ, Candelaria D, Tait MA, Norman R, King MT. Health-related quality of life of Australians during the 2020 COVID-19 pandemic: a comparison with pre-pandemic data and factors associated with poor outcomes. Quality of Life Research, 2022.

ANZCTR trial registration: ACTRN12621001240831

Appendix 1. Summary of lockdown dates, infection rates and restrictions during our data collection period: **21 October to 10 November 2020*** and during the first Australian wave of COVID-19

*most data obtained by 4 November 2020.

Table A1.1. Number of New Cases during our survey period: 21 October to 10 November 2020

	**Cases acquired locally**	**Cases acquired Internationally and in quarantine**	**Total**
NSW	21	104	125
VIC	30	0	30
QLD	0	15	15
SA	0	34	34
WA	4	36	40
ACT	1	0	1
TAS	0	0	0
NT	0	0	0
**Australia**	**56**	**189**	**245**

Table A1.2. Summary of lockdown and restrictions <u>during our survey period: 21 October – 10 November 2020</u>, including the easing of restrictions from Melbourne/Victoria's second wave of COVID-19 infections (30 June – November 2020)*.

	VIC-Metro	VIC-Rural	NSW	QLD	TAS	WA	SA	NT	ACT
Face masks M=Mandatory SR=Strongly recommended	Mandatory	Mandatory	Strongly recommended.	Strongly recommended	Strongly recommended	Strongly recommended	Strongly recommended	Strongly recommended	Strongly recommended
Curfew	Not in place	Not in place	Not in place	Not in place	Not in place	Not in place	Not in place	Not in place	Not in place
House visitors (number per day, distance from the household rule and exclusions)	<u>From 18 Oct:</u><br>1 nominated visitor if living alone/single parent<br>Respite care for people with needs allowed<br><br><u>From 27 Oct:</u><br>Up to 2 from the same household, 25km rule applies	Up to 2 from different households	<u>From 16 Oct:</u><br>Up to 50 at a time, and no more than 30 strongly recommended if residence has no outdoor area	Up to 50	Up to 40	No limit, 2 sq m rule applies	Up to 10, 4 sq m rule applies	No limit, 1.5 sq m rule applies	No limit
Outdoor gathering	<u>From 18 Oct:</u><br>Up to 10 from a maximum of 2 households<br><br><u>From 27 Oct:</u><br>Up to 10 from any number of households	Up to 70	<u>From 23 Oct:</u><br>Up to 30 (previously 20)	Up to 50 (does not apply to businesses operating with a COVID-safe plan	Up to 1,000, 2 sq m rule applies	No limit, 2 sq m rule applies	Up to 50, 4 sq m rule applies	No limit, 1.5 sq m rule applies<br>Gatherings of >100 will require the completion of a COVID-safe plan.	Up to 500, 2 sq m rule applies

	VIC-Metro	VIC-Rural	NSW	QLD	TAS	WA	SA	NT	ACT
Hospitality venues (restaurants, cafes, pubs)	From 18 Oct:<br>Take-away and delivery only<br><br>From 27 Oct:<br>Up to 10 per table/group,, Up to 20 for indoor and 50 for outdoor venues.	Up to 10 per indoor space with a maximum of 40 for indoor venues.<br>2 and 4 sq m rules apply.<br>Take-away only for people from metropolitan Melbourne	From 16 Oct:<br>2 sq m rule for outdoor areas (previously 4 sq m rule)<br>Functions of up to 300 people per venue, subject to a COVID-safe plan<br><br>From 23 Oct:<br>Up to 30 per booking/table(previously 10)	No limit, 2 sq m rule and COVID-safe plan applies	Up to 250 for indoor and 1,000 for outdoor venues, 2 sq m rule applies	No limit, 2 sq m rule and COVID-safe plan applies	Up to 100 per venue, and up to 10 per booking, 4 sq m rule applies, seated dining only	No restrictions, COVID-safe plan applies	Up to 500 per venue, 4 sq m rule applies
Intra-state travel	From 18 Oct:<br>25 km rule applies, (previously 5 km) Travel to regional Victoria only for permitted purposes, even if within 25 kms<br><br>From 27 Oct:<br>Travel to regional Victoria only for permitted purposes, even if within 25 kms<br>From 8 Nov:<br>Allowed	No information	No restrictions	No restrictions	No restrictions	No restrictions	No restrictions	No restrictions	No restrictions

	VIC-Metro	VIC-Rural	NSW	QLD	TAS	WA	SA	NT	ACT
Inter-state travel	From 18 Oct:<br>Not allowed, stay local, 25 km rule applies<br><br>From 27 Oct:<br>Not allowed, stay local, 25 km rule applies	From 18 Oct:<br>Not allowed, stay local, 25 km rule applies	Border to VIC closed (re-opened 23 November).	Open to all states except VIC<br>QLD Border Declaration Pass required if have been in a hotspot in the last 14 days, or overseas and did not fly into QLD when arrived in Australia.	Open to all states except VIC<br>Registration via the Tas e-Travel system required at least three days before entering TAS	Open to all states, except VIC<br>G2G PASS registration and declaration process required	Open to all states, except VIC<br>A cross-border travel registration required	Open to all states, except VIC<br>A border entry form and declaration required up to 72 hours before arrival.	Open to all states, except VIC<br>An online declaration required
Wedding or funeral attendance<br><br>*Note: Would only affect a person who had to cancel a wedding, or missed a wedding or funeral*	From 18 Oct:<br>**Weddings**:<br>In outdoor public spaces only with up to 5 people (including the couple and two witnesses but not the celebrant)<br>**Funerals**:<br>Up to 10 people (not including people conducting the funeral)<br><br>From 27 Oct:<br>**Weddings:**	No Info	From 23 October:<br>Up to 300 for weddings and 100 for funerals, 4 and 2 sq m rule apply<br>Up to 20 people in the wedding party can dance on the dance floor, but only for members of the wedding party.<br>A record of names and contact details of each guest must be kept.	Up to 200 for weddings and funerals at professional venues.<br>Up to 30 for private weddings, including the wedding party and celebrant if there is no COCIV-safe plan.<br>A record of names and contact details of each guest must be kept for 56 days.	Up to 250 for indoor, and up to 1,000 for outdoor spaces, 2 sq m rule applies	No limit, 2 sq m rule applies	Up to 150 for weddings and 50 for funerals, 4 sq m rule apply for both	No limit, but a COVID-safe plan required for more than 100 people	Up to 500 for both weddings and funerals, 4 sq m rule apply for both

	VIC-Metro	VIC-Rural	NSW	QLD	TAS	WA	SA	NT	ACT
	Up to 10 people outdoors (including the couple and two witnesses but not including the celebrant and 1 photographer)<br>**Funerals:**<br>Up to 20 people both indoors and outdoors (not including people conducting the funeral)<br><br><u>From 8 November:</u> Up to 50 people outdoors (not including people conducting the funeral)								
Place of worship<br><br>*Note: Would only affect a person who wished to attend a religious service*	<u>From 18 Oct:</u><br>Closed<br>Up to 5 people, plus 1 faith leader for outdoor gatherings (not ceremonies), no sharing of food, drink or other	Up to 20 people plus 1 faith leader for outdoor religious gatherings (previously 10), no sharing of food, drink, crockery, utensils, vessels	<u>From 23 October:</u><br>Up to 300, 4 sq m rule applies, even if men and women are in separate areas. Reconsider activities that might spread the virus – singing and passing collection baskets. The use of face masks is	Up to 50, 4 sq m rule applies (2 sq m rule for venues less than 200 sq m)	Up to 250, 2 sq m rule applies	No limit, 2 sq m rule applies	Up to 100, 4 sq m rule applies	No limit, 1.5 sq m rule applies	Up to 25, excluding those conducting the service

	VIC-Metro	VIC-Rural	NSW	QLD	TAS	WA	SA	NT	ACT
	items by participants<br><br>From 27 Oct:<br>Up to 10 plus 1 faith leader for indoor ceremonies, with cleaning requirements between services<br>Up to 20, plus 1 faith leader for outdoor religious gatherings, no sharing of food, drink or other items by participants.	or other equipment by participants	strongly recommended for all services.						
Schools	From 12 Oct:<br>Staged return to onsite learning.<br><br>From 27 Oct:<br>Open	No Info (same as Metro Melb)	From 5 Oct: Resumes with strict COVID-safe plan<br><br>High school formals permitted from November 12.	Open	Open	Open	Open	Open	Open
Salons, spas and other beauty services	From 18 Oct:<br>Beauty/personal care services open, face mask mandatory for the duration of service	No Info (same as Metro Melb)	Open	Open	Open	Open	Open	Open	Open

	VIC-Metro	VIC-Rural	NSW	QLD	TAS	WA	SA	NT	ACT
Cinemas, entertainment venues, museums, libraries and open houses	From 18 Oct:<br>All venues closed<br><br>From 27 Oct:<br>Outdoor venues and spaces open with density quotients, patron caps and COVID-safe plans<br><br>From 8 November:<br>Electronic gaming allowed subject to patron and time limits	Up to 20 for indoor libraries and toy libraries, 4 sq m rule applies	Open, 4 sq m rule applies, and a Covid-19 safety plan.<br>Up to 50% capacity and allocated seats for large venues	No limit, with a COVID-safe plan.<br>Face masks mandatory	Up to 250 for indoor and 1,000 for outdoor facilities, 2 sq m rule applies permitting	No limit, 2 sq m rule applies<br>Up to 50% capacity cap on major sport and entertainment venues.<br>Large scale, multi-stage music festivals prohibited	No limit, 4 sq m rule applies	No limit	Up to 50% capacity for each venue, allocated seats, 4 sq m rule applies
Gym and exercise activities	From 18 Oct:<br>**Outdoor recreation:**<br>25 km rule applies, can use outdoor sport and recreation facilities<br>Up to 2 people per trainer for outdoor personal training	Up to 20 in the pool or 4 sq m rule (whichever is smaller)<br>One parent/guardian/carer per child is permitted for supervision<br>Swimming classes can resume	Up to 20 per class, 4 sq m rule applies, excluding staff<br>Up to 500 for community sporting competitions and training, 4 sq m rule applies, excluding staff	Up to 50 indoor (2 or 4 sq m rule applies depending on size of venue)<br>No limit for outdoor non-contact sport and group training and boot camps, 4 sq m rule applies	Up to 250 for indoor venues, 2 sq m rule applies<br>Up to 1,000 for outdoor gathering<br>Full contact training and full competition sport (contact and non-contact) is	No limits, 2 sq m rule applies<br>Gyms can operate unstaffed but must undergo regular cleaning.<br>Contact sport and training can also recommence, and playgrounds,	No limits for gyms, recreation centres, trampoline and play cafes, as well as outdoor fitness activities, such as boot camps and personal training sessions, 4 sq	No limits for gyms, fitness studios and indoor training activities such as Cross Fit, as well as team sports such as football, basketball, soccer and netball.	Up to 100 for indoor gyms and fitness centres, 4 sq m rule applies.

	VIC-Metro	VIC-Rural	NSW	QLD	TAS	WA	SA	NT	ACT
	Outdoor pools open, indoor swimming pools open for one-on-one hydrotherapy sessions with a health professional where clinically indicated<br>No group sessions<br>**Exercise:**<br>Up to 10 from 2 households for outdoor exercise<br><br><u>From 27 Oct:</u><br>**Sport and physical recreation for adults:**<br>Minimum number required to play game for outdoor non-contact sports,<br>Up to 10 for outdoor fitness and fitness classes				allowed, as is sharing equipment, change rooms and other facilities	outdoor gym equipment and skate parks can be used.	m rule applies<br>Indoor play centres, amusement parks and arcades remain closed, as well as community or club sports fixtures and trainings, whether indoors or outdoors<br>Swimming only for fitness or rehabilitation		

	VIC-Metro	VIC-Rural	NSW	QLD	TAS	WA	SA	NT	ACT
	(excluding the trainer).<br><br>From 8 November:<br>Up to 10 people in a space and 20 in a venue, subject to density limits<br><br>**Indoor pools (including swimming classes):**<br>Open for one-on-one hydrotherapy sessions with a health professional, carer or support person<br>No group sessions<br><br>From 8 November:<br>Up to 20 per venue<br><br>**Outdoor swimming pools:**								

	VIC-Metro	VIC-Rural	NSW	QLD	TAS	WA	SA	NT	ACT
	Up to 50 or density limits, other than for exclusive use by a single school at any one time for education purposes or community sport								
Restrictions prior to our data collection following second wave of infections in Victoria	2 Aug 2020 – 13 Sept 2020 VIC a State of Disaster. **Stage 4 restrictions for Metro Melbourne**:<br>- curfew (8pm-5am)<br>- compulsory face masks<br>- four essential reasons for leaving the house<br>- exercise: 2 hours max, with 1 other person only<br>- shopping: 1 person per household<br>- 5km rule<br>- Remote learning	**Stage 3 restrictions from <u>5 August 2020</u> for Regional VIC**<br>- Four reasons to be out<br>- Compulsory face masks<br>- Cafes and restaurants takeaway only<br>- Nonessential shops to close<br>- No community sport<br>- Remote learning							

***** During our data collection period (21 October to 10 November 2020), Public Health Orders remained within the remit of each state and territory; therefore, the type and duration of restrictions varied widely across the country, as summarised in this document.

Key dates:

13 Sept 2020 – 'Roadmap' for reducing restrictions in Victoria commences (see Victoria's roadmap for reopening – How we live in Metropolitan Melbourne (amaze.org.au) for details)

19 Oct 2020 – Restrictions are significantly eased in Victoria, with further easing on 27 October as cases continue to be less than 5

26 Oct 2020 – VIC records zero new cases and deaths for the first time since 9 June

Table A1.3. Summary of restrictions during the first wave of COVID-19 in Australia (early 2020)

	VIC-Metro	VIC-Rural	NSW	QLD	TAS	WA	SA	NT	ACT
First reported case	25 Jan 2020		25 Jan 2020	29 Jan 2020	2 Mar 2020	21 Feb 2020	1 Feb 2020	4 Mar 2020	12 Mar 2020
National lockdown	23 Mar 2020		23 Mar 2020	23 Mar 2020	23 Mar 2020	23 Mar 2020	23 Mar 2020	23 Mar 2020	23 Mar 2020
*Start of easing hard lockdown**	11 May 2020<br>(7 weeks)<br>- up to 10 outdoors<br>- up to 5 for visiting at someone's home<br>- up to 10 for weddings<br>- up to 20 for funerals held indoors and 30 for outdoors<br>- resumption of some outdoor recreational activities		1 May 2020<br>(6 weeks)<br>- up to two adults and dependent children to visit another household	15 May 2020<br>(8 weeks)<br>- Stage 1 reopening:<br>- up to 10 in a public space<br>- recreational travel of a radius of up to 150km from the person's home for day trips<br>- re-opening of libraries, playground equipment, skate parks and outdoor gyms, with up to 10 people at a time	11 May 2020<br>(7 weeks)<br>- 20 people to attend funerals<br>- people to visit those living in residential aged care once a week<br>- TasTAFE campuses and training facilities to open for small groups of students	27 April 2020<br>(5 weeks)<br>- Stage 1 reopening<br>- up to 10 for indoor and outdoor non-work gatherings	11 May 2020<br>(7 weeks)<br>- Stage 1 reopening	1 May 2020<br>(6 weeks)<br>- Stage 1 reopening<br>Stage one adjustments for simple and safe outdoor activities where physical distancing can be maintained at all times. Including:<br>Personal gatherings including outdoor weddings and funerals.<br>Playgrounds, parks and campgrounds outside biosecurity areas.<br>Public swimming pools, lagoons and water parks.<br>Outdoor sports where physical distancing can be maintained including golf and tennis as well as training outdoors.	15 May 2020<br>(8 weeks)<br>- cafés and restaurants to seat up to ten people

	VIC-Metro	VIC-Rural	NSW	QLD	TAS	WA	SA	NT	ACT
								Go fishing, boating, and sailing with other people, real estate open house inspections and auctions, and gatherings in homes.	
Further easing of restriction	26 May 2020<br>- phased return to the classroom		11 May 2020<br>- return to face-to-face teaching<br>- increased retail activity<br><br>15 May 2020<br>- up to 10 for outdoor gatherings<br>- up to 10 for cafés and restaurants<br>- up to 5 visitors to a household<br><br>1 June 2020<br>- travel to regional NSW<br>- up to 50 for places of worship funeral<br>- camping grounds and	12 June 2020<br>- Stage 2 reopening<br><br>10 July<br>- borders open, except for VIC	17 June 2020<br>- 20 visitors to a home in addition to household members<br>- groups of 20 to participate in social sports activities and<br>- 20 attendees per pool<br><br>13 July 2020<br>- Stage 3 reopening<br><br>24 July 2020<br>- borders reopen	18 May 2020<br>- Stage 2 reopening<br>- up to 20 for indoor and outdoor non-work gatherings<br>- people encouraged to return to work, unless they are unwell or vulnerable<br>- up to 20 for cafés and restaurants<br><br>6 June 2020<br>- Stage 3 – up to 100 for non-work indoor and outdoor<br>- up to 300 per venue<br>- food businesses and	15 May 2020<br>- opening of campsites and national parks with amended services to ensure social distancing<br><br>1 June 2020<br>- Stage 2 reopening: up to 80 for pubs, gyms, cinemas, places of worship, beauty salons and other sites<br><br>29 June 2020<br>- Step 3 reopening	15 May 2020<br>- Stage 2 Reopening<br>Stage two activities (safer indoor activities for less than two hours), such as:<br>Shopping centre food courts.<br>Restaurants, cafes, and bars for the consumption of food – excluding gaming areas.<br>Organised outdoor training activities for sport teams without physical contact.<br>Beauty therapy salons for non-facial services such as nails, massage and tanning.	18 May 2020<br>- staged return to on-campus schooling<br><br>29 May 2020<br>- up to 50 people for funeral<br>- up to 20 for cafés, bars, restaurants and clubs<br>- beauty therapy businesses to reopen<br><br>19 June 2020<br>- cafés, restaurants, bars and other licenced venues to seat up to 100<br>- contact sports to start

	VIC-Metro	VIC-Rural	NSW	QLD	TAS	WA	SA	NT	ACT
			caravan parks to open<br><br>1 July<br>- gyms and fitness studios to reopen (up to 10 per class and 100 people in an indoor venue)<br>- children's sport and community sports competition for people aged up to 18 years to resume<br>- up to 10 for tattoo and massage parlours			licensed premises to operate with seated service<br>- alcohol to be served without a meal at licensed premises<br><br>27 June 2020<br>- Stage 4 reopening			full training from 19 June to start their transition back to competitions from 10 July.

* Defined as the day any form of restriction was eased.

Sources:

NSW: https://www.health.nsw.gov.au/news/Pages/2020-nsw-health.aspx

VIC: https://www.theguardian.com/australia-news/datablog/ng-interactive/2020/oct/20/coronavirus-australia-map-cases-covid-19-tracking-stats-live-data-update-by-state-suburb-postcode-how-many-new-active-case-numbers-today-statistics-corona-deaths-death-toll

QLD: https://www.theguardian.com/australia-news/datablog/ng-interactive/2020/oct/20/coronavirus-australia-map-cases-covid-19-tracking-stats-live-data-update-by-state-suburb-postcode-how-many-new-active-case-numbers-today-statistics-corona-deaths-death-toll

SA: https://www.covid-19.sa.gov.au/home/dashboard/dashboard-table-data#covid-19-daily

WA: https://covidlive.com.au/wa

ACT:
https://app.powerbi.com/view?r=eyJrIjoiZTY4NTI1NzQtYTBhYy00ZTY4LTk3NmQtYjBjNzdiOGMzZjM3IiwidCI6ImI0NmMxOTA4LTAzMzQtNDIzNi1iOTc4LTU4NWVlODhlNDE5OSJ9

NT: https://coronavirus.nt.gov.au/;

https://newsroom.nt.gov.au/mediaRelease/33205

Appendix 2. Original COVID-19 measures: Items and scoring

COVID Impact Index

1. What impact has COVID-19 had on your time availability?
   (1=much worse; 5=much better)
2. What impact has COVID-19 had on your job security?
   (1=much worse; 5=much better)
3. What impact has COVID-19 had on your financial situation?
   (1=much worse; 5=much better)
4. What impact has COVID-19 had on your household/family responsibilities (e.g. daily chores, parenting, schooling children)?
   (1=much more responsibility; 5=much less responsibility)
5. What impact has COVID-19 had on your extended family responsibilities (caring for people not living in your household e.g., shopping, medical care, emotional support)?
   (1=much more responsibility; 5=much less responsibility)
6. What impact has COVID-19 had on your nutrition/diet?
   (1=much worse; 5=much better)
7. What impact has COVID-19 had on your amount of physical activity (intentional and/or incidental)?
   (1=much less; 5=much more)
8. What impact has COVID-19 had on your quality of sleep?
   (1=much worse; 5=much better)
9. What impact has COVID-19 had on your family relationships?
   (1=much worse; 5=much better)
10. What impact has COVID-19 had on your mental health?
    (1=much worse; 5=much better)
11. What impact has COVID-19 had on your physical health?
    (1=much worse; 5=much better)
12. What impact has COVID-19 had on your quality of social connections (including via digital means)?
    (1=much worse; 5=much better)
13. What impact has COVID-19 had on your alcohol consumption?
    (1=much less; 5=much more)
14. What impact has COVID-19 had on your substance use?
    (1=much less; 5=much more)
15. What impact has COVID-19 had on your living situation (e.g., living space)?
    (1=much less comfortable; 5=much more comfortable)
16. What impact has COVID-19 had on how lonely you feel?
    (1=much less; 5=much more)
17. What impact has COVID-19 had on time spent on hobbies/leisure activities?
    (1=much less; 5=much more)
18. What impact has COVID-19 had on your daily routine?
    (1=much worse; 5=much better)
19. What impact has COVID-19 had on your work/study productivity?
    (1=much worse; 5=much better)

Scoring (mean score calculated for each composite using the below items):
Job security, finances, routine, mental health & relationships: Items 2, 3, 9, 10, 12, 15, 18, 19 (higher score = more positive impact)
Physical health/health behaviours: Items 6, 7, 8, 11 (higher scores = more positive impact)
Family responsibilities: Items 4, 5 (higher scores = less responsibilities)

Alcohol/substance use: Items 13, 14 (higher scores = more use)
Loneliness & time: Items 1, 16, 17 (higher scores = more)

Reference: Kleitman, S., Fullerton, D. J, Zhang, L. M. & Aidman, E. Validating cognitive fitness constructs as predictors of self-reported psychological well-being and its recovery after the first COVID-19 lockdown in Australia (submitted in PLOS ONE October 2021).

COVID Worry Scale
To what extent do the following statements describe how you feel NOW:
1=never; 4=always

1. I am nervous when I think about the pandemic
2. I am calm and relaxed when I think about the pandemic*
3. I am worried about my health due to COVID-19
4. I am worried about the health of my family members due to COVID-19
5. I am stressed about leaving my house
6. I am worried about someone I love dying due to COVID-19 related reasons
7. I am worried about returning to face-to-face interactions
8. I am stressed about taking public transport
9. I am concerned about the possibility of another wave of COVID-19 in the city/region where I live, or where my friends/family live
10. I am concerned about the possibility of another lockdown in the city/region where I live, or where my friends/family live
11. I am worried about losing money
12. I am worried about becoming unemployed
13. I am worried about global economic recession
14. I am worried about political systems failing
15. I am worried about my financial situation
16. I am worried about the Australian economy
17. I am worried about society and social liberties degrading
18. I am worried about grocery stores running out of food and/or other supplies
19. I am worried about pharmacies running out of medicines/essential health supplies
20. I am worried about our healthcare system being overloaded
21. I am worried about the healthcare system not being able to protect me or my loved ones

**Reverse scored*

Scoring:
Personal/Family Concerns: Items 1, 2, 3, 4, 5, 6, 7, 8, 9, 10
Personal Financial Concerns: Items 11, 12, 15
Economy/Liberties Concerns: Items 13, 14, 16, 17
Infrastructure/Supplies Concerns: Items 18, 19, 20, 21
Higher scores = greater worries.

The original version of this scale (first 5 items) was taken from Kleitman et al. (2021). The scale was revised with additional items added for this and previous research to reflect ongoing changes related to the pandemic, and relevant manuscripts regarding this measure and its validation are currently undergoing preparation.

Reference: Kleitman, S., Fullerton, D. J., Zhang, L., Blanchard, M. D., Lee, J., Stankov, L., & Thompson, V. (2021). To comply or not comply? A latent profile analysis of behaviours and attitudes during the COVID-19 pandemic. PLOS ONE, 16(7): https://doi.org/10.1371/journal.pone.0255268

Possible Impacts of COVID

***What positive impacts did you experience as a result of the first wave of COVID-19 restrictions (March-May 2020)?**

Please select ONLY if this is a POSITIVE impact for you.

Select all that apply:

- Time saved from not needing to commute to work
- Extra time with immediate family/people you live with
- More communications with extended family (virtual communication)
- Neighbourhood initiatives such as the rainbow trail (rainbows in windows),"going on a bear hunt" (finding teddy bears in neighbour's windows) or similar
- Time to complete household chores (indoors & outdoors)
- Awareness about risk-reducing behaviours (e.g. Hand washing, use of hand sanitiser, not shaking hands, maintaining social distancing)
- Opportunities to assist members of the community who are less fortunate with donations or charity
- Extra time to focus on health (exercise, diet)
- Reducing environmental footprint (using your car less, not traveling overseas)
- Health care appointments were conducted via telehealth
- Mental health appointments were conducted via telehealth

Scoring: A total score 'positive impacts' was calculated by summing the number of items selected.

***What negative impacts have you experienced as a result of the COVID pandemic in Australia?**

Please select ONLY if this is a NEGATIVE impact for you.

Select all that apply:

- Recreational activities were cancelled
- Health care appointments and treatments were cancelled or postponed
- Mental health appointments were cancelled or postponed
- Health care appointments were conducted via telehealth
- Mental health appointments were conducted via telehealth
- Isolated from a family member who usually resides in the same house as you
- Isolated from a family member who does not live with you
- Loss of time to myself during commute to work
- Too much time with immediate family/people you live with

Scoring: A total score 'negative impacts' was calculated by summing the number of items selected.

***What other impacts have you experienced as a result of the COVID pandemic in Australia?**

Select all that apply:

- Kept children home from school/day care
- Switched to online shopping
- Learned how to use new software or apps, e.g. Zoom, Microsoft Teams, What's App etc.

Scoring: A total score 'other impacts' was calculated by summing the number of items select

Attitude and Motivation Towards Compliance
Please rate the extent to which you agree or disagree with each statement:
1=strongly disagree; 5=strongly agree

1. I am willing to comply with the current COVID-19 rules and recommendations
2. I am aware of the current COVID-19 rules and recommendations
3. I adhere to the current COVID-19 rules and recommendations
4. Should another lockdown be needed, I will follow the rules
5. If we have another lockdown, I will make sure my household follow the rules
6. If we have another lockdown, I can promptly adapt to the required lifestyle again
7. If we have another lockdown, I will be willing to adapt my behaviours
8. If we have another lockdown, I will break the rules*
9. Having a second lockdown will violate my rights*

**reverse scored*

Scoring: Mean score of all items. Higher scores = greater willingness and motivation towards compliance with COVID-19 restrictions.

Reference: Kleitman, S., Fullerton, D., Zhang, L. M. & Aidman, E. Validating cognitive fitness constructs as predictors of compliance attitudes after the first COVID-19 lockdown in Australia (in preparation).

Appendix 3. Data quality checking protocol

Summary

- Based on the advice of Wardropper et al (2021), our data quality checking protocol included seven **quality checks** to identify poor quality data/responders:
    - **Inconsistent responses**: Six logic checks based on inconsistent responses to related items as listed in Table A3.1.
    - **Speedy completers:** Overall survey completion time less than 7.79 minutes, which is equivalent to 30% of the median completion time and spending 1.68 seconds per mandatory item (i.e. excluding sections that only some participants were required to complete, therefore the time spent per question is likely to have been even less for some participants.)

      This formula is used by the online survey provider, Survey Engine, to identify participants considered to be "speeders", or participants who completed the survey too quickly to comprehend and reflect on the questions adequately.
- Figure A3.1 outlines our data quality checking process and reasons for participant exclusions and Table A3.2 presents number of participants failing each check.
    - 99 people were excluded for failing 2 or more data quality checks, including completion time and/or logic checks (see Table A3.3).
    - 10 additional participants did not fail any logic checks but were excluded on excessively fast completion time alone as they were deemed to have completed the survey too quickly to comprehend and reflect on the questions adequately (1.68 seconds per mandatory item).
- In total 109 participants were excluded on the basis of having provided poor quality data, resulting in a final analysis sample of 1898 participants.

Reference: Wardropper et al, Conducting conservation social science surveys online 2021. Conservation Biology. 2021;1–9. DOI: 10.1111/cobi.13747

Figure A3.1. Participant inclusions and exclusions.

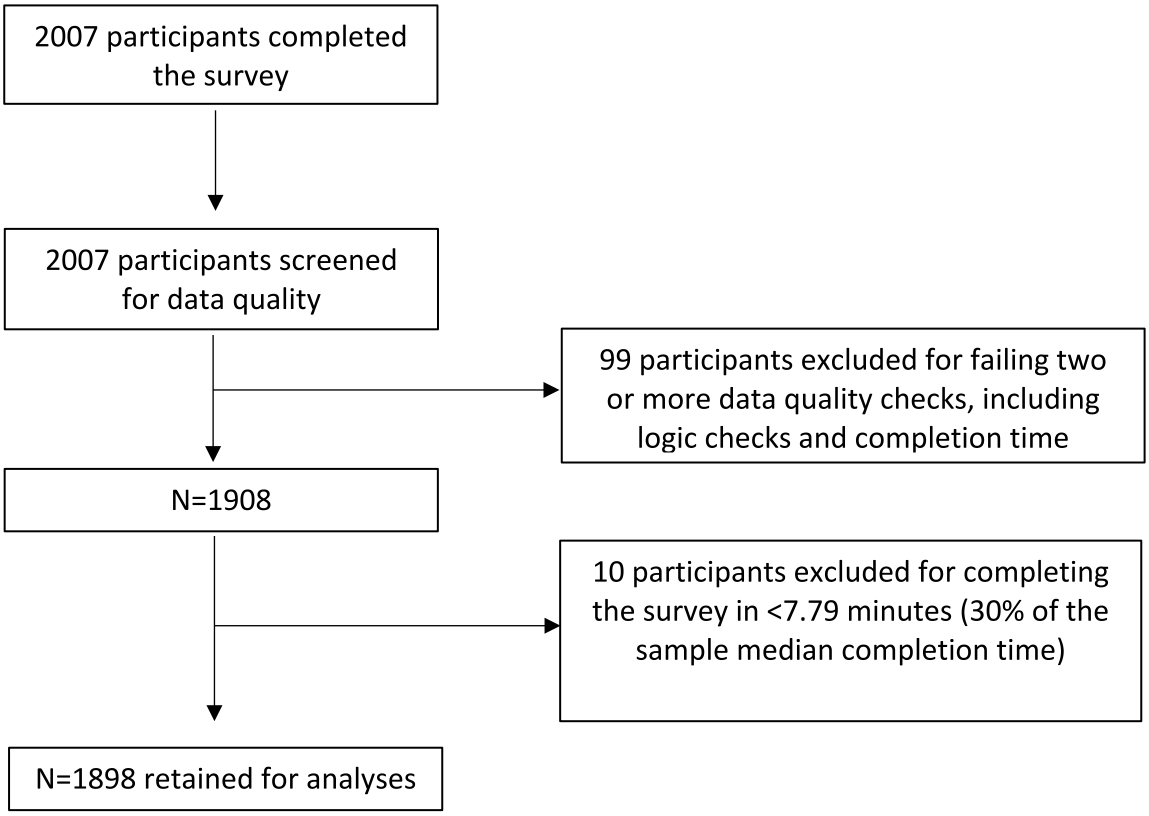

Table A3.1. Logic Checks for Inconsistent Responses

Scale	Variable 1 Item(s)	Variable 1 Scoring	Variable 2 Item(s)	Variable 2 Scoring	Criteria for Inconsistent Responses
QLQ-C30	Average of QLQC30 items 6 to 28, e.g.<br>6) Were you limited in doing either your work or other daily activities?<br>12) Have you felt weak?	(1) *not at all*<br>(2) *a little*<br>(3) *quite a bit*<br>(4) *very much*	Average of QLQC30 items:<br>29) How would you rate your overall health during the past week?<br>30) How would you rate your overall quality of life during the past week?	(1) *very poor* to (7) *excellent*	• ≥3 on variable 1 AND ≥5.5 on variable 2; OR<br>• ≤2 on variable 1 AND ≤1.5 on variable 2
COVID Worry	I am nervous when I think about the pandemic	(1) *never*<br>(2) *sometimes*<br>(3) *often*<br>(4) *always*	I am calm and relaxed when I think about the pandemic	(1) *never*<br>(2) *sometimes*<br>(3) *often*<br>(4) *always*	• 1 on variable 1 AND 1 on variable 2; OR<br>• 4 on variable 1 AND 4 on variable 2
COVID Behaviours (a)	I stay at home	(1) *never*<br>(2) *sometimes*<br>(3) *often*<br>(4) *always*	I attend social gatherings	(1) *never*<br>(2) *sometimes*<br>(3) *often*<br>(4) *always*	• 4 on variable 1 AND 4 on variable 2; OR<br>• 4 on variable 1 AND 3 on variable 2
COVID Behaviours (b)	I stay at home	(1) *never*<br>(2) *sometimes*<br>(3) *often*<br>(4) *always*	I avoid crowded places	(1) *never*<br>(2) *sometimes*<br>(3) *often*<br>(4) *always*	• 4 on variable 1 AND 1 on variable 2
Brief COPE Inventory	Using alcohol or other drugs to make myself feel better	(1) *I haven't been doing this at all*<br>(2) *I've been doing this a little bit*	Using alcohol or other drugs to help me get through it	(1) *I haven't been doing this at all*<br>(2) *I've been doing this a little bit*	• 1 on variable 1 AND 3 on variable 2; OR<br>• 1 on variable 1 AND 4 on variable 2; OR

		(3) *I've been doing this a medium amount*<br>(4) *I've been doing this a lot*		(3) *I've been doing this a medium amount*<br>(4) *I've been doing this a lot*	• 3 on variable 1 AND 1 on variable 2; OR<br>• 4 on variable 1 AND 1 on variable 2
Compliance	Should another lockdown be needed, I will follow the rules	(1) *strongly disagree*<br>(2) *disagree*<br>(3) *neither agree nor disagree*<br>(4) *agree*<br>(5) *strongly agree*	If we have another lockdown, I will break the rules	(1) *strongly disagree*<br>(2) *disagree*<br>(3) *neither agree nor disagree*<br>(4) *agree*<br>(5) *strongly agree*	• 1 on variable 1 AND 1 on variable 2; OR<br>• 1 on variable 1 AND 2 on variable 2; OR<br>• 2 on variable 1 AND 1 on variable 2; OR<br>• 2 on variable 1 AND 2 on variable 2; OR<br>• 3 on variable 1 AND 3 on variable 2; OR<br>• 3 on variable 1 AND 4 on variable 2; OR<br>• 4 on variable 1 AND 3 on variable 2; OR<br>• 4 on variable 1 AND 4 on variable 2

Table A3.2. Number of participants flagged for each data quality check

Data Quality Check		Frequency
**Completion time <7.79 minutes** (1.68 seconds per mandatory item)		25
**Data inconsistency checks**	QLQ-C30	38
	COVID Worry	104
	COVID Behaviours (a)	57
	COVID Behaviours (b)	11
	Brief COPE Inventory	61
	Compliance	307

Table A3.3. Frequencies of total data quality checks failed

Number of flags (i.e. data quality checks failed)	Frequency (N=2007)
0	1548
1	360
2	67
3	19
4	13

Appendix 4. Potential explanatory variables for all 15 QLQ-C30 domains and the GHQ regression models

Explanatory variable	Description	Levels/allowable range
1. COVID Impact Index Factor 1: better job security/finances/routine/mental health & relationships	Mean score, continuous	Range 1-5
2. COVID Impact Index Factor 2: better physical health/activity/sleep/nutrition	Mean score, continuous	Range 1-5
3. COVID Impact Index Factor 3: less family responsibilities	Mean score, continuous	Range 1-5
4. COVID Impact Index Factor 4: more alcohol/substance use	Mean score, continuous	Range 1-5
5. COVID Impact Index factor 5: lonely/ more time	Mean score, continuous	Range 1-5
6. Number of positive impacts	Summed, continuous	Range 0-12
7. Number of negative impacts	Summed, continuous	Range 0-10
8. Number of other impacts	Summed, continuous	Range 0-4
9. Health care services worker	Dichotomous	1= yes, 0=no
10. Change in work hours	Hours worked in an average week in the 6 months before COVID, MINUS hours worked in an average week now.	Range 0-168
11. Attitude and Motivation towards compliance	Mean score, continuous	Range 1-5
12. COVID Worry scale, factor 1. Personal/Family Concerns (items 1 to 10).	Continuous	Range 1-4
13. COVID Worry scale, factor 2. Personal Financial Concerns (items 11, 12, 15).	Continuous	Range 1-4
14. COVID Worry scale, factor 3. Economy/Liberties Concerns (items 13, 14, 16, 17).	Continuous	Range 1-4
15. COVID Worry scale, factor 4. Infrastructure/Supplies Concerns (items 18 to 21).	Continuous	Range 1-4
16. Cared for children who were usually at school/day care whilst working	Dichotomous	1= yes, 0=no
17. Sex	Dichotomous	0=male, 1=female
18. Age	Continuous	18-99 years
19. Living with partner	Dichotomous.<br><br>Yes = Married (in a registered marriage)<br>OR never married but living with someone in a relationship;<br><br>No = Separated but not divorced<br>OR Divorced<br>OR Widowed<br>OR Never married and not living with someone in a relationship	1= yes, 0=no
20. Location within Australia: Melbourne, VIC – Y/N	Dichotomous	1= yes, 0=no
21. Location within Australia: VIC regional – Y/N	Dichotomous	1= yes, 0=no
22. Location within Australia: NSW/QLD – Y/N	Dichotomous	1= yes, 0=no

Please note that scoring for the COVID Impact Index, Attitude and Motivation towards compliance and COVID Worry scales are explained in Appendix 2.

Appendix 5. Regression Analyses

Table A5.1 Means, standard deviations, skewness and reliability estimates for continuous variables included in the regression models

	Mean (SD)	Skewness	α
Dependent Variables			
Global Health/QOL	62.92 (21.88)	-.50	.82
Physical Functioning	84.75 (20.61)	-1.45	.85
Role Functioning	82.10 (24.75)	-1.31	.75
Emotional Functioning	71.80 (26.36)	-.84	.89
Cognitive Functioning	82.91 (22.47)	-1.44	.69
Social Functioning	85.00 (24.77)	-1.70	.56
Fatigue	24.41 (23.38)	.97	.77
Nausea/Vomiting	8.40 (17.52)	2.36	.70
Pain	21.82 (27.73)	1.24	.85
Dyspnoea[a]	14.21 (25.04)	1.82	-
Insomnia[a]	29.42 (32.66)	.84	-
Appetite Loss[a]	12.54 (23.26)	1.96	-
Constipation[a]	12.12 (24.12)	2.08	-
Diarrhoea[a]	9.92 (21.05)	2.29	-
Financial Difficulties[a]	12.26 (24.79)	2.14	-
General health question[a]	2.85 (1.02)	.08	-
COVID-19 Impact Index			
Job security, finances, routine, mental health & relationships	2.69 (.55)	-.30	.80
Physical health/sleep/nutrition	2.76 (.68)	-.19	.72
Family responsibilities	2.64 (.71)	-.34	.60
Alcohol/substance use	2.82 (.75)	-.82	.59
Loneliness/boredom[b]	3.11 (.62)	-.05	.19
COVID-19 Worry Scale			
Personal Financial Concerns	2.20 (.91)	.42	.90
Personal/Family Concerns	2.38 (.69)	.31	.90
Economy/Liberties Concerns	2.49 (.78)	.17	.83
Infrastructure/Supplies Concerns	2.06 (.83)	.60	.84
Compliance Attitudes	4.10 (.84)	-.73	.92

Note. SD = standard deviation. α = Cronbach's alpha.

a. Alpha values are not given for these variables as they consist of only one item.
b. We note that the loneliness/boredom COVID-19 impact dimension has a low alpha value. This is to be expected given that this score is comprised of three more diverse items (impact on loneliness, time spent on hobbies, and time availability). Factor analysis was run to determine dimensionality of the scale; thus the scoring of these subscales was kept consistent with the results of the factor analysis.

Table A5.2 Pearson bivariate correlations between all continuous explanatory variables in the models

	**1**	**2**	**3**	**4**	**5**	**6**	**7**	**8**	**9**	**10**	**11**	**12**	**13**	**14**
1 COVID Impact Index: Better job security, finances, routine, mental health & relationships														
2 COVID Impact Index: Better physical health/sleep/nutrition	.60**													
3 COVID Impact Index: Less family responsibilities	.35**	.29**												
4 COVID Impact Index: More alcohol/substance use	.05*	-.02	.04											
5 COVID Impact Index: More lonely/ bored	.24**	.22**	.13**	.17**										
6 COVID Worry Scale: Personal Financial Concerns	-.43**	-.23**	-.30**	.02	.00									
7 COVID Worry Scale: Personal/Family Concerns	-.25**	-.21**	-.30**	-.01	.04	.56**								
8 COVID Worry Scale: Economy/Liberties Concerns	-.27**	-.17**	-.21**	-.05*	.03	.53**	.54**							
9 COVID Worry Scale: Infrastructure/Supplies Concerns	-.20**	-.18**	-.30**	-.05*	-.03	.53**	.69**	.56**						
10 Number of positive impacts	.03	.06**	-.13**	-.09**	.13**	.08**	.21**	.12**	.09**					
11 Number of negative impacts	-.28**	-.19**	-.25**	.02	.03	.26**	.28**	.22**	.24**	.37**				
12 Number of other impacts	-.13**	-.11**	-.35**	.00	.03	.27**	.30**	.15**	.23**	.37**	.41**			
13 Attitudes and motivation towards compliance	.11**	.08**	.09**	-.03	.11**	-.15**	.05*	-.04	-.10**	.18**	-.12**	-.07**		
14 Change in work hours (before first lockdown – now)	-.21**	-.07**	-.09**	-.02	0.03	.19**	.05*	.08**	.03	.09**	.09**	.11**	-.01	
15 Age	.08**	.07**	.24**	-.08**	.05*	-.37**	-.17**	.00	-.23**	-.02	-.14**	-.34**	.38**	-.07**

Note. * $p < .05$; ** $p < .01$.

Table A5.3 Pearson bivariate correlations between continuous explanatory variables and dependent variables

	Global health/QOL	Physical Functioning	Role Functioning	Emotional Functioning	Cognitive Functioning	Social Functioning	Fatigue	Nausea/ Vomiting	Pain	Dyspnoea	Insomnia	Appetite Loss	Constipation	Diarrhoea	Financial Difficulties	GHQ
COVID Impact Index: Better job security, finances, routine, mental health & relationships	.32**	.02	.17**	.36**	.23**	.17**	-.17**	-.06*	-.06*	-.03	-.23**	-.13**	-.07**	-.01	-.14**	-.23**
COVID Impact Index: Better physical health/sleep/nutrition	.32**	.09**	.17**	.30**	.23**	.19**	-.21**	-.08**	-.09**	-.08**	-.27**	-.14**	-.05*	-.06*	-.16**	-.29**
COVID Impact Index: Less family responsibilities	.06*	.02	.16**	.18**	.13**	.12**	-.11**	-.12**	-.02	-.05*	-.16**	-.13**	-.05*	-.06**	-.16**	-.01
COVID Impact Index: More alcohol/substance use	-.03	.00	-.03	-.07**	-.06*	-.05*	.03	.04	.00	.04	.06*	.04	.03	.03	.05*	-.01
COVID Impact Index: More lonely/ bored	-.01	-.01	.04	-.07**	-.03	-.04	.06*	-.01	.07**	.01	.05*	.01	.05*	.00	.03	.01
COVID Worry Scale: Personal Financial Concerns	-.21**	-.06**	-.20**	-.45**	-.28**	-.19**	.22**	.21**	.08**	.11**	.26**	.24**	.18**	.18**	.28**	.08**
COVID Worry Scale: Personal/Family Concerns	-.24**	-.22**	-.28**	-.45**	-.28**	-.28**	.29**	.21**	.18**	.21**	.26**	.25**	.21**	.13**	.29**	.16**
COVID Worry Scale: Economy/Liberties Concerns	-.17**	-.12**	-.16**	-.31**	-.18**	-.19**	.19**	.10**	.11**	.14**	.18**	.12**	.09**	.04	.16**	.11**
COVID Worry Scale: Infrastructure/Supplies Concerns	-.20**	-.24**	-.27**	-.35**	-.25**	-.30**	.25**	.28**	.17**	.22**	.20**	.26**	.19**	.16**	.30**	.08**
Number of positive impacts	.12**	.05*	-.02	-.04	-.03	.00	.00	.00	-.03	-.01	.03	.02	.06**	-.01	.02	-.09**
Number of negative impacts	-.15**	-.14**	-.23**	-.27**	-.24**	-.20**	.18**	.16**	.15**	.14**	.21**	.16**	.14**	.14**	.23**	.05*
Number of other impacts	.01	.00	-.15**	-.19**	-.12**	-.08**	.08**	.14**	-.02	.04	.12**	.10**	.11**	.10**	.11**	-.10**
Attitudes and motivation towards compliance	.14**	.16**	.16**	.13**	.16**	.15**	-.07**	-.27**	-.03	-.16**	-.07**	-.18**	-.18**	-.21**	-.19**	.07**
Change in work hours (before first lockdown – now)	-.03	.09**	-.01	-.09**	-.07**	.01	.01	-.01	-.05*	-.02	.04	.04	.04	-.00	.01	.01
Age	.01	-.01	.15**	.21**	.17**	.09**	-.09**	-.30**	.07**	-.07**	-.09**	-.24**	-.18**	-.22**	-.17**	.19**

Note. * $p < .05$; ** $p < .01$.

Table A5.4. Spearman correlations between dichotomous explanatory variables and dependent variables

	Global health/QOL	Physical Functioning	Role Functioning	Emotional Functioning	Cognitive Functioning	Social Functioning	Fatigue	Nausea/ Vomiting	Pain	Dyspnoea	Insomnia	Appetite Loss	Constipation	Diarrhoea	Financial Difficulties	GHQ
Healthcare services worker (1=yes; 0=no)	.07**	.07**	.04	.01	.04	.05*	-.04	-.03	-.06**	-.06*	-.03	-.06**	.00	-.02	-.04	-.05*
Cared for children who were usually at school/day-care whilst working (1=yes; 0=no)	-.01	.01	-.07**	-.07**	-.05*	-.03	.01	.06**	.01	-.01	.05*	.05*	.05*	.03	.05*	-.01
Living in Melbourne Metropolitan (1=yes; 0=no)	.01	.05*	-.03	-.03	.03	.05*	-.04	-.07**	-.08**	-.05*	-.04	-.08**	-.03	-.02	-.07**	-.04
Living in Regional Victoria (1=yes; 0=no)	-.05*	-.02	-.07**	-.04	-.05*	-.03	.04	.02	.06**	.02	.04	.03	.02	.04	.06*	.02
Living in NSW/QLD (1=yes; 0=no)	.05*	-.03	.03	.09**	.05*	.01	-.01	-.01	-.02	-.00	-.01	-.02	-.01	-.05*	-.01	-.00
Living with partner (1=yes; 0=no)	.11**	.07**	.08**	.07**	.05*	.05*	-.07**	-.05*	-.05*	-.05*	-.05*	-.07**	-.01	-.01	-.04	-.06**
Sex (0=male; 1=female)	-.02	-.03	.00	-.15**	-.04	-.01	.11**	.03	.05	-.02	.11**	.02	.04	-.03	-.04	.09**

*Note. * p < .05; ** p* <.01.

Tables A5.5-5.8 report the results of regression analyses predicting general health question (GHQ) and EORTC QLQ-C30 scores. The results discussed in this section are only those significant at the adjusted significance level. Those significant at p < .05 are marked in the tables.

GHQ: The model predicting GHQ was statistically significant, explaining 18.7% of variance, F(22,1871) = 19.54, p < .001. The strongest predictor of higher GHQ scores was a more negative impact of COVID-19 on physical health and activity. Impact of COVID-19 on work, mental health and social life was also a negative predictor. Personal/family concerns and age were small positive predictors. Lastly, fewer positive impacts of COVID was a weak predictor.

Global health/QOL: The model predicting global health/QOL was statistically significant, explaining 22.5% of variance, F(22,1871) = 24.75, p < .001. The strongest predictor of better global health/QOL life was less worry about oneself and one's family being infected with COVID-19. A more positive impact of COVID on work, mental health, and social life also predicted better global health/QOL, as was the case for impact on physical health. Less family responsibilities negatively predicted global health/QOL;, as well as more loneliness and time; however, this shared a negligible correlation with the outcome variable (r = -.01, p = .81) indicating a possible suppressor effect. A greater number of positive COVID-related impacts and lower number of negative impacts also contributed to global health/QOL scores, as did more positive attitudes towards compliance. For demographics, living with a partner and being younger were the only significant predictors. However, age shared no correlation with global health/QOL (r = .02, p = .52) indicating a suppressor effect, thus this finding should be interpreted with caution.

Physical functioning: The model predicting physical functioning was significant, accounting for 15.7% of variance, F(22, 1871) = 15.82, p < .001. Personal/family concerns (negatively) and compliance attitudes (positively) were the strongest predictors of better physical functioning. Lower concerns about infrastructure and supplies, lower financial concerns, less negative impacts and living with a partner were also significant predictors of better physical functioning. Age was also significant, though had a very low zero-order correlation with the dependent variable, indicating a possible suppression effect.

Role functioning: The model predicting role functioning was significant, explaining 15.2% of variance, F(22, 1871) = 15.25, p < .001. Lower worry about oneself and family, and lack of infrastructure and supplies were significant predictors of better role functioning. Fewer negative impacts, higher scores on compliance attitudes, and living with a partner were also significant predictors.

Emotional functioning: The model predicting emotional functioning was significant, explaining 35.6% of variance, F(22,1871) = 46.96, p <.001. The strongest predictor was lower personal/family concerns, followed by lower financial concerns. Three COVID impact domains were significant, such that better work life, mental health, and social life; better physical health and activity; and less loneliness and time as a result of the pandemic predicted better emotional functioning. Fewer negative impacts, more positive compliance attitudes, and being male were also weaker predictors. Lastly, living in Metropolitan Melbourne and NSW or Queensland were also weak predictors; however, Melbourne did not share a significant correlation with the dependent variable (r = -.03, p = .24), indicating suppression.

Cognitive functioning: The model predicting cognitive functioning was significant, accounting for 17.3% of variance, F(22, 1871) = 17.79, p <.001. Impact of COVID on physical health and compliance attitudes were significant positive predictors. Personal/family concerns and number of negative impacts were significant negative predictors.

Social functioning: The model predicting social functioning was significant, capturing 16.4% of variance, F(22, 1871) = 16.63, p <.001. Significant predictors were positive impact on physical health, having less time and loneliness due to COVID-19, lower personal/family concerns, lower concerns about supplies and infrastructure, fewer negative impacts, positive compliance attitudes, and living in Metropolitan Melbourne. However, it should be noted that impact on loneliness and

time shared a trivial bivariate correlation with the dependent variable (*r* = -.04, *p* = .11), indicating a possible suppression effect.

Fatigue: The model predicting fatigue was statistically significant, capturing 15.5% of variance, F(22, 1871) = 15.59, *p* <.001. The strongest predictors were personal/family concerns and worse physical health and activity due to COVID. Greater negative impacts and being female also predicted fatigue. Not living in Metropolitan Melbourne was also significant.

Nausea: The model predicting nausea was significant, explaining 18.8% of total variance, F(22, 1871) = 19.65, *p* <.001. Concerns about self/family and infrastructure/supplies, number of negative impacts, negative attitudes towards compliance, and being younger were significant predictors of nausea. Not living in Metropolitan Melbourne was also significant.

Pain: The model predicting pain was significant, explaining 10.7% of total variance, F(22, 1871) = 10.19, *p* <.001. Personal/family concerns and experiencing more negative COVID impacts were the strongest predictors. Being older, having fewer positive impacts, having more time availability and loneliness, and not living in Metropolitan Melbourne were also significant; however positive impacts had a non-significant bivariate correlation with pain (*r* = -.03, *p* =.20), suggesting a suppression effect.

Dyspnoea: The model predicting dyspnoea was significant, explaining 11.1% of variance, F(22, 1871) = 10.59, *p* <.001. Personal/family concerns, infrastructure/supplies concerns, greater negative impacts, and more negative attitudes towards compliance were significant predictors.

Insomnia: The model predicting insomnia was significant, capturing 16.6% of the total variance, F(22, 1871) = 16.97, *p* <.001. Worse physical health due to the pandemic, greater concerns about personal finances and self/family, a greater number of negative impacts, and being female were significant predictors of insomnia. Experiencing more loneliness and boredom and not living in Metropolitan Melbourne were also significant, though living in Melbourne showed negligible zero-order correlation with the outcome (*r* = -.04, *p* = .07) suggesting suppressor effects.

Appetite loss: The model predicting appetite loss was significant, explaining 15.4% of variance, F(22, 1871) = 15.54, *p* <.001. Personal/family concerns, more negative attitudes towards compliance, not living in Metropolitan Melbourne, and being younger were significant predictors of appetite loss.

Constipation: The model predicting constipation was significant, explaining 10.7% of total variance, F(22, 1871) = 10.16, *p* <.001. The only significant predictors were personal/family concerns and more negative compliance attitudes.

Diarrhoea: The model predicting diarrhoea was significant, explaining 10.9% of total variance, F(22, 1871) = 10.38, *p* <.001. Better work life/mental health/social life, financial concerns, and negative COVID impacts were significant positive predictors. Compliance attitudes and age were significant negative predictors. However impact on work/mental health/social life shared no correlation with diarrhoea (*r* = -.01, *p* =.67), indicating a suppression effect.

Financial difficulties: The model predicting financial difficulties was significant, explaining 17.9% of total variance, F(22, 1871) = 18.55, *p* <.001. All dimensions of COVID-19 worry were significant predictors, positively, except for economic concerns which was negative. Worse physical health and activity, more negative impacts, and negative attitudes towards compliance were also significant predictors. Living in Metropolitan Melbourne was also significant.

Table A5.5. Summary of regression analyses predicting general health question scores, global QOL/health, physical functioning, and role functioning

	General Health Question				Global QOL/Health				Physical Functioning				Role Functioning			
**Predictor**	**B**	**SE**	***β***	***p***	**B**	**SE**	***β***	***p***	**B**	**SE**	***β***	***p***	**B**	**SE**	***β***	***p***
COVID Impact Index: better job security/finances/routine/mental health/relationships	-.18	.06	-.10	.001**	6.68	1.18	.17	<.001**	-2.88	1.16	-.08	.01*	1.20	1.39	.03	.39
COVID Impact Index: better physical health/sleep/nutrition	-.36	.04	-.24	<.001**	6.12	.85	.19	<.001**	2.24	.84	.07	.01*	1.61	1.01	.04	.11
COVID Impact Index: less family responsibilities	.08	.04	.05	.03*	-2.66	.75	-.09	<.001**	-.10	.73	-.03	.17	.89	.88	.03	.31
COVID Impact Index: more alcohol/substance use	-.02	.03	-.02	.48	-.19	.62	-.01	.76	.19	.61	.01	.75	-.93	.73	-.03	.20
COVID Impact Index: more lonely/ bored	.10	.04	.06	.018	-3.17	.79	-.09	<.001**	-.80	.80	-.02	.31	.65	.94	.02	.49
Personal financial Concerns	.03	.04	.03	.36	-.51	.73	-.02	.48	2.25	.72	.10	.003**	.86	.87	.03	.32
Personal/Family Concerns	.28	.05	.19	<.001**	-6.98	1.01	-.22	<.001**	-6.64	.99	-.22	<.001**	-6.30	1.19	-.18*	<.001**
Economy/Liberties Concerns	-.04	.04	-.03	.25	1.03	.78	.04	.19	1.25	.77	.05	.10	1.22	.92	.04	.19
Infrastructure/Supplies Concerns	-.04	.04	-.03	.29	-.53	.83	-.02	.52	-4.23	.82	-.17	<.001**	-3.56	.98	-.12	<.001**
Number of positive impacts	-.04	.01	-.09	<.001**	1.20	.24	.12	<.001**	.66	.23	.07	.004*	.49	.28	.05	.08
Number of negative impacts	.03	.02	.04	.11	-1.18	.34	-.09	<.001**	-1.75	.34	-.13	<.001**	-2.23	.41	-.14	<.001**
Number of other impacts	-.09	.03	-.08	.003*	1.38	.65	.06	.03	.93	.64	.04	.14	-.68	.77	-.02	.38
Attitude and Motivation towards compliance – total score	.01	.03	.01	.67	3.47	.61	.13	<.001**	4.83	.60	.20	<.001**	3.35	.72	.11	<.001**
Healthcare services worker (1=yes; 0=no)	-.12	.10	-.02	.25	3.28	2.18	.03	.13	5.40	2.14	.05	.01*	5.00	2.57	.04*	.05*
Change in work hours (before first lockdown – now)	-.00	.00	-.01	.74	.02	.04	.01	.62	.12	.04	.06	.004*	.06	.05	.03	.24
Cared for children who were usually at school/day-care whilst working	.10	.09	.03	.24	-.95	1.82	-.01	.60	-.99	1.79	-.01	.58	1.22	2.16	.01	.57
Location within Australia: Melbourne VIC metro	-.17	.06	-.07	.01*	3.12	1.30	.06	.02*	2.56	1.27	.05	.05*	-.51	1.53	-.01	.74
Location within Australia: VIC regional	-.08	.06	-.03	.22	.71	1.28	.01	.58	.27	1.26	.01	.83	-2.34	1.51	-.04	.12
Location within Australia: NSW/QLD	-.02	.06	-.01	.79	1.91	1.28	.04	.13	-.99	1.25	-.02	.43	-1.56	1.51	-.03	.30
Living with partner (1=yes; 0=no)	-.08	.05	-.04	.07	3.56	.94	.08	<.001**	3.07	.93	.07	<.001**	4.17	1.11	.08	<.001**
Age	.01	.00	.21	<.001**	-.10	.03	-.09	.001**	-.13	.03	-.12	<.001**	.03	.04	.02	.45
Sex (1=female; 0=male)	.12	.04	.06	.01*	.30	.91	.01	.75	.12	.90	.00	.90	.04	1.08	.00	.97

Note. B = unstandardized regression coefficient. SE = standard error of B. *β* = standardized regression coefficient. *$p < .05$; **p < adjusted significance level.

Table A5.6. Summary of regression analyses predicting emotional functioning, cognitive functioning, social functioning, and fatigue

	Emotional Functioning				Cognitive Functioning				Social Functioning				Fatigue			
**Predictor**	**B**	**SE**	**β**	***p***	**B**	**SE**	**β**	***p***	**B**	**SE**	**β**	***p***	**B**	**SE**	**β**	***p***
COVID Impact Index: better job security/finances/routine/mental health/relationships	7.26	1.29	.15	<.001**	1.94	1.25	.05	.12	2.97	1.38	.07	.03	-.58	1.31	-.01	.66
COVID Impact Index: better physical health/sleep/nutrition	4.22	.93	.11	<.001**	4.22	.90	.13	<.001**	3.19	1.00	.09	.001**	-4.81	.95	-.14	<.001**
COVID Impact Index: less family responsibilities	-2.06	.82	-.06	.01	-1.05	.79	-.03	.18	-.32	.88	-.01	.72	.35	.83	.01	.67
COVID Impact Index: more alcohol/substance use	-1.51	.68	-.04	.03	-1.03	.65	-.03	.11	-1.53	.72	-.05	.04	.29	.69	.01	.68
COVID Impact Index: more lonely/ bored	-4.23	.87	-.10	<.001**	-2.21	.84	-.06	.01*	-3.13	.93	-.08	<.001**	2.96	.88	.08	<.001**
Personal financial Concerns	-5.10	.80	-.18	<.001**	-1.89	.78	-.08	.02*	1.70	.86	.06	.05	.53	.82	.02	.52
Personal/Family Concerns	-10.94	1.11	-.29	<.001**	-5.30	1.07	-.16	<.001**	-6.68	1.19	-.19	<.001**	6.65	1.12	.20	<.001**
Economy/Liberties Concerns	-.13	.86	-.00	.88	.75	.83	.03	.36	.47	.92	.01	.61	.20	.87	.01	.82
Infrastructure/Supplies Concerns	.51	.91	.02	.57	-1.20	.88	-.04	.17	-4.81	.98	-.16	<.001**	1.68	.92	.06	.07
Number of positive impacts	.55	.26	.05	.03	.20	.25	.02	.42	.45	.28	.04	.11	-.58	.26	-.06	.03*
Number of negative impacts	-1.68	.38	-.10	<.001**	-1.95	.36	-.14	<.001**	-1.89	.40	-.12	<.001**	1.61	.38	.11	<.001**
Number of other impacts	-.13	.71	.00	.86	1.02	.69	.04	.14	.73	.76	.03	.34	-.34	.72	-.01	.63
Attitude and Motivation towards compliance – total score	2.37	.67	.08	<.001**	3.00	.65	.11	<.001**	3.75	.72	.13	<.001**	-1.71	.68	-.06	.01*
Healthcare services worker (1=yes; 0=no)	-1.00	2.39	-.01	.67	1.59	2.31	.01	.49	2.91	2.56	.02	.26	-3.48	2.43	-.03	.15
Change in work hours (before first lockdown – now)	.02	.05	.01	.72	-.04	.05	-.02	.38	.09	.05	.04	.09	-.06	.05	-.03	.18
Cared for children who were usually at school/day-care whilst working	2.19	2.00	.02	.27	-.79	1.93	-.01	.68	1.45	2.14	.02	.50	-3.52	2.03	-.04	.08
Location within Australia: Melbourne VIC metro	4.44	1.42	.07	.002**	3.77	1.37	.07	.01*	4.74	1.52	.08	.002**	-4.21	1.45	-.08	.004*
Location within Australia: VIC regional	3.96	1.40	.07	.005	1.11	1.35	.02	.41	1.77	1.50	.03	.24	-.82	1.43	-.02	.56
Location within Australia: NSW/QLD	4.27	1.40	.07	.002**	2.25	1.35	.04	.10	.80	1.50	.01	.59	.09	1.42	.00	.95
Living with partner (1=yes; 0=no)	2.12	1.03	.04	.04	.98	1.00	.02	.33	2.15	1.11	.04	.05	-2.59	1.05	-.05	.01*
Age	.08	.04	.06	.02	.06	.03	.05	.06	-.02	.04	-.01	.61	.00	.04	.00	.96
Sex (1=female; 0=male)	-5.43	1.00	-.10	<.001**	-.96	.97	-.02	.32	1.00	1.08	.02	.35	4.25	1.02	.09	<.001**

Note. B = unstandardized regression coefficient. SE = standard error of B. β = standardized regression coefficient. **p* < .05; ***p* < adjusted significance level.

Table A5.7. Summary of regression analyses predicting nausea, pain, dyspnoea, and insomnia

	Nausea				Pain				Dyspnoea				Insomnia			
**Predictor**	**B**	**SE**	**β**	***p***	**B**	**SE**	**β**	***p***	**B**	**SE**	**β**	***p***	**B**	**SE**	**β**	***p***
COVID Impact Index: better job security/finances/routine/mental health/relationships	1.47	.97	.05	.13	1.28	1.60	.03	.42	3.52	1.44	.08	.02*	-1.68	1.82	-.03	.36
COVID Impact Index: better physical health/sleep/nutrition	-.63	.70	-.02	.37	-2.58	1.16	-.06	.03*	-2.14	1.04	-.06	.04*	-9.00	1.31	-.19	<.001**
COVID Impact Index: less family responsibilities	.03	.61	.00	.96	.87	1.01	.02	.39	.75	.91	.02	.41	-1.21	1.15	-.03	.29
COVID Impact Index: more alcohol/substance use	.44	.50	.02	.39	-.30	.84	-.01	.72	1.10	.75	.03	.14	1.57	.95	.04	.10
COVID Impact Index: more lonely/ bored	.38	.65	.01	.56	3.48	1.08	.08	.001**	.27	.97	.01	.78	4.49	1.23	.08	<.001**
Personal financial Concerns	.13	.60	.01	.82	-.49	1.00	-.02	.62	-1.33	.90	-.05	.14	3.86	1.13	.11	<.001**
Personal/Family Concerns	2.99	.83	.12	<.001**	6.60	1.37	.17	<.001**	6.48	1.24	.18	<.001**	7.43	1.56	.16	<.001**
Economy/Liberties Concerns	-1.48	.64	-.07	.02*	-1.34	1.06	-.04	.21	.27	.95	.01	.78	-.04	1.20	.00	.97
Infrastructure/Supplies Concerns	3.28	.68	.16	<.001**	3.20	1.13	.10	.01*	3.27	1.02	.11	.001**	-1.26	1.28	-.03	.33
Number of positive impacts	-.23	.19	-.03	.23	-1.03	.32	-.08	.001**	-.47	.29	-.04	.10	-.43	.36	-.03	.24
Number of negative impacts	.92	.28	.08	.001**	2.86	.47	.16	<.001**	1.92	.42	.12	<.001**	2.14	.53	.10	<.001**
Number of other impacts	.17	.53	.01	.75	-1.73	.88	-.06	.05*	-.49	.79	-.02	.54	.28	1.00	.01	.78
Attitude and Motivation towards compliance – total score	-4.02	.50	-.19	<.001**	-1.93	.83	-.06	.02*	-4.87	.75	-.16	<.001**	-2.32	.94	-.06	.01
Healthcare services worker (1=yes; 0=no)	-2.55	1.78	-.03	.15	-5.99	2.96	-.04	.04*	-4.85	2.67	-.04	.07	1.18	3.37	.01	.73
Change in work hours (before first lockdown – now)	-.04	.04	-.02	.28	-.13	.06	-.05	.02*	-.04	.05	-.02	.49	-.07	.07	-.02	.29
Cared for children who were usually at school/day-care whilst working	-1.82	1.49	-.03	.22	.52	2.48	.00	.83	-2.92	2.23	-.03	.19	-.96	2.82	-.01	.73
Location within Australia: Melbourne VIC metro	-4.46	1.06	-.11	<.001**	-5.76	1.76	-.09	.001**	-4.28	1.59	-.07	.01*	-6.97	2.01	-.09	<.001**
Location within Australia: VIC regional	-2.42	1.05	-.06	.02*	.65	1.74	.01	.71	-1.65	1.57	-.03	.29	-1.09	1.98	-.01	.58
Location within Australia: NSW/QLD	-2.27	1.05	-.06	.03*	-1.57	1.74	-.02	.37	-.85	1.56	-.01	.59	.10	1.97	.00	.96
Living with partner (1=yes; 0=no)	.20	.77	.01	.79	-3.41	1.28	-.06	.01*	-2.69	1.15	-.05	.02*	-2.64	1.46	-.04	.07
Age	-.16	.03	-.17	<.001**	.20	.04	.13	<.001**	.05	.04	.03	.23	.06	.05	.03	.27
Sex (1=female; 0=male)	.92	.75	.03	.22	1.09	1.24	.02	.38	-1.45	1.12	-.03	.20	5.18	1.42	.08	<.001**

Note. B = unstandardized regression coefficient. SE = standard error of B. *β* = standardized regression coefficient. **p* < .05; ***p* < adjusted significance level.

Table A5.8. Summary of regression analyses predicting appetite loss, constipation, diarrhoea, and financial difficulties

	Appetite Loss				Constipation				Diarrhoea				Financial Difficulties			
**Predictor**	**B**	**SE**	**β**	**p**	**B**	**SE**	**β**	**p**	**B**	**SE**	**β**	**p**	**B**	**SE**	**β**	**p**
COVID Impact Index: better job security/finances/routine/mental health/relationships	.43	1.31	.01	.74	-.44	1.39	-.01	.75	4.94	1.21	.13	<.001**	1.86	1.37	.04	.18
COVID Impact Index: better physical health/sleep/nutrition	-2.75	.94	-.08	.004*	-.04	1.01	.00	.97	-1.93	.88	-.06	.03*	-3.17	.99	-.09	.001**
COVID Impact Index: less family responsibilities	-.14	.83	.00	.87	2.27	.88	.07	.01*	.54	.77	.02	.48	-1.17	.87	-.03	.18
COVID Impact Index: more alcohol/substance use	.69	.68	.02	.31	.48	.73	.01	.51	-.01	.63	.00	.99	.93	.72	.03	.20
COVID Impact Index: more lonely/ bored	1.39	.88	.04	.11	1.85	.94	.05	.05*	.36	.82	.01	.66	2.27	.92	.06	.01
Personal financial Concerns	1.03	.81	.04	.21	.75	.87	.03	.38	2.69	.76	.12	<.001**	3.31	.85	.12	<.001**
Personal/Family Concerns	5.43	1.12	.16	<.001**	6.83	1.19	.20	<.001**	1.99	1.04	.07	.06	5.97	1.18	.17	<.001**
Economy/Liberties Concerns	-1.98	.86	-.07	.02*	-2.05	.92	-.07	.03*	-2.29	.80	-.08	.004	-2.79	.91	-.09	.002**
Infrastructure/Supplies Concerns	2.76	.92	.10	.003*	1.29	.98	.04	.19	1.50	.86	.06	.08	3.40	.97	.11	<.001**
Number of positive impacts	.02	.26	.00	.93	.40	.28	.04	.15	-.35	.24	-.04	.15	-.26	.27	-.02	.34
Number of negative impacts	1.03	.38	.07	.01*	.89	.41	.06	.03	1.49	.36	.11	<.001**	2.32	.40	.15	<.001**
Number of other impacts	-1.26	.72	-.05	.08	-.20	.77	-.01	.80	.00	.67	.00	.99	-1.24	.75	-.04	.10
Attitude and Motivation towards compliance – total score	-3.36	.68	-.12	<.001**	-4.82	.72	-.17	<.001**	-3.66	.63	-.15	<.001**	-4.31	.71	-.15	<.001**
Healthcare services worker (1=yes; 0=no)	-6.21	2.42	-.06	.01*	.33	2.58	.00	.90	-1.61	2.25	-.02	.47	-1.27	2.54	-.01	.62
Change in work hours (before first lockdown – now)	.02	.05	.01	.72	.03	.05	.01	.54	-.03	.04	-.01	.53	-.05	.05	-.02	.33
Cared for children who were usually at school/day-care whilst working	-.51	2.02	-.01	.80	-.50	2.16	-.01	.82	-1.76	1.88	-.02	.35	-1.63	2.13	-.02	.44
Location within Australia: Melbourne VIC metro	-5.86	1.44	-.11	<.001**	-3.61	1.53	-.07	.02*	-2.23	1.34	-.05	.10	-5.04	1.51	-.09	<.001**
Location within Australia: VIC regional	-1.29	1.42	-.02	.36	-.98	1.51	-.02	.52	-.83	1.32	-.02	.53	-1.52	1.49	-.03	.31
Location within Australia: NSW/QLD	-1.92	1.42	-.04	.17	-.77	1.51	-.01	.61	-2.84	1.32	-.06	.03*	-1.94	1.49	-.03	.19
Living with partner (1=yes; 0=no)	-1.64	1.04	-.03	.12	.65	1.11	.01	.56	.18	.97	.00	.85	-1.16	1.10	-.02	.29
Age	-.18	.04	-.13	<.001**	-.10	.04	-.08	.01*	-.12	.03	-.10	<.001**	.00	.04	.00	.93
Sex (1=female; 0=male)	.56	1.02	.01	.58	1.30	1.08	.03	.23	-1.04	.94	-.02	.27	-2.22	1.07	-.04	.04

Note. B = unstandardized regression coefficient. SE = standard error of B. β = standardized regression coefficient. *$p < .05$; **$p <$ adjusted significance level.

Appendix6. Demographic characteristics, continued…

Question	Level	Frequency	2015/16 Sample % *(or mean [SD])*	2020 Sample % (or *mean [SD]*)	Population % (or mean [SD])[a]	Statistic [b,d]	*p* value[d]
Country of Birth[e]	Australia	1461	74.1	77.0	71.7	X^2 = 65.10	<.001
	England	92	-	4.8	4.2		
	New Zealand	36	-	1.9	2.4		
	India	48	-	2.5	2.1		
	Italy	11	-	.6	.8		
	Vietnam	15	-	.8	1.0		
	Philippines	33	-	1.7	1.1		
	Other	202	-	10.6	16.7		
Australian Citizenship[e]	Yes	1637	-	86.2	82.4	X^2 = 19.55	<.001
Employment Status[e]	Employed	1092	-	57.5	56.1	X^2 = 257.83	<.001
	Unemployed	227	-	12.0	4.1		
	Not in the labor force	579	-	30.5	33.1		
	Not stated	-	-	-	6.7		
Work Industry[e]	Agriculture, Forestry, Fishing	78	-	7.1	2.5		
	Mining	13	-	1.2	1.7		
	Manufacturing	55	-	5.0	6.4		
	Electricity, Gas, Water and Waste Services	15	-	1.4	1.1		
	Construction	73	-	6.7	8.5		
	Wholesale Trade	19	-	1.7	2.9		
	Retail Trade	98	-	9.0	9.9		
	Accommodation and Food Services	72	-	6.5	6.9		
	Transport, Postal and Warehousing	42	-	3.9	4.7		
	Information Media and Telecommunications	39	-	3.6	1.7		
	Financial and Insurance Services	52	-	4.7	3.6		
	Rental, Hiring and Real Estate Services	17	-	1.6	1.7		
	Professional, Scientific and Technical Services	90	-	8.3	7.3		
	Administrative and Support Services	93	-	8.5	3.4		
	Public Administration and Safety	33	-	3.0	6.7		
	Education and Training	134	-	12.3	8.7		
	Health Care and Social Assistance	96	-	8.8	12.6		
	Arts and Recreation Services	36	-	3.3	1.7		
	Other Services	89	-	8.2	3.7		
Chronic Conditions	Asthma, emphysema, or chronic bronchitis	277	13.5	14.6	n/a		
	Arthritis or rheumatism	334	19.9	17.6	20.5	X^2 = 9.64	.002
	Cancer diagnosed in last 3 years	68	2.8	3.6	n/a		
	Diabetes	192	9.3	10.1	6.2	X^2 = 48.87	<.001
	Digestive problems (e.g. stomach ulcer, colitis, or gallbladder disease)	112	7.1	5.9	7.4	X^2 = 5.98	.01
	Heart trouble (e.g. angina, congestive heart failure, or coronary artery disease)	118	5.8	6.2	n/a		
	HIV illness or AIDS	19	0.4	1.0	n/a		
	Kidney disease	39	1.6	2.1	1.2	X^2 = 12.17	<.001
	Liver problems (e.g. cirrhosis)	29	1.6	1.5	n/a		
	Stroke	16	1.3	.8	.8	X^2 = .21	.65[3]
	None of these	1131	59	59.6	n/a		

a. Population values for Country of Birth were obtained from the Australian Bureau of Statistics 2016 Census (note, this data was not limited to those aged 18 and over). Prevalence of chronic conditions were derived from the Australian Bureau of Statistics National Health Survey, published December, 2018: https://www.abs.gov.au/statistics/health/health-conditions-and-risks/national-health-survey-first-results/latest-release. Population data for other chronic conditions was not directly comparable in format.
b. The chi-squared goodness of fit test was used to compare observed distributions to those expected based on Australian population data.
c. Indicates sample is not statistically significantly different from the Australian general population.
d. Comparisons are between 2020 sample and population data[a.]
e. Data not available from 2015/16 reference sample: Mercieca-Bebber, R., et al. (2019). "The EORTC Quality of Life Questionnaire for cancer patients (QLQ-C30): Australian general population reference values." Med J Aust 210(11): 499-506.
